# Effective options for addressing air quality– related environmental public health burdens in Saudi Arabia

**DOI:** 10.1016/j.heliyon.2022.e10335

**Published:** 2022-08-19

**Authors:** Jared Woollacott, Wael Alsufyani, Robert H. Beach, Laura T. R. Morrison, Alison Bean de Hernández, Severin Rakic, Mashael AlOmran, Reem F. Alsukait, Christopher H. Herbst, Salem AlBalawi

**Affiliations:** a RTI International, 3040 E Cornwallis Rd, Research Triangle Park, North Carolina, 27709, USA; b Public Health Authority of Saudi Arabia, Riyadh, Saudi Arabia; c Health, Nutrition and Population Global Practice, The World Bank, Riyadh Country Office, Saudi Arabia; d Community Health Sciences Department, College of Applied Medical Sciences, King Saud University, Riyadh, Saudi Arabia; e The World Bank, Washington, D.C., USA

**Keywords:** Air pollution, Public health, Air quality, Mitigation, Adaptation, Environment

## Abstract

Air pollution poses major disease burdens globally and accounts for approximately 10% of deaths annually through its contribution to a variety of respiratory, cardiovascular, and other diseases. The burden of disease is particularly acute in Saudi Arabia, where a mix of anthropogenic and natural sources of air pollution threatens public health. Addressing these burdens requires careful study of the costs and effectiveness of available technologies and policies for reducing emissions (mitigation) and avoiding exposure (adaptation). To help evaluate these options, we conduct a semi-systematic literature review of over 3,000 articles published since 2010 that were identified by searches of literature focused on pollution mitigation and pollution adaptation. We identify a wide variety of effective mitigation and adaptation technologies and find that cost-effectiveness information for policy design is highly variable in the case of mitigation, both within and across pollution source categories; or scarce, in the case of adaptation. While pollution control costs are well studied, policy costs differ; these may vary more by location because of factors such as technology operating conditions and behavioral responses to adaptation initiatives, limiting the generalizability of cost-effectiveness information. Moreover, potential cost advantages of multipollutant control policies are likely to depend on the existing mix of pollution sources and controls. While the policy literature generally favors more flexible compliance mechanisms that increase the cost of polluting to reflect its costs to society, important policy design factors include policy co-benefits, distributional concerns, and inter-regional harmonization. In addition to these key themes, we find that further study is needed both to improve the availability of cost information for adaptation interventions and to localize technology and policy cost estimates to the Saudi context.

## Highlights

• Literature review of environmental public health technology and policy options.
• Air pollution mitigation options have considerable cost variation.
• Air pollution adaptation options lack thorough cost effectiveness evaluation.
• Policy effectiveness will depend heavily on local conditions and design.
• Saudi Arabia requires a mix of mitigation and adaptation public health options.

## 1 Introduction

Air pollution remains a major contributor to the global burden of disease, accounting for approximately 10% of annual deaths globally (WHO, 2016). Ambient concentrations of fine particulate matter (PM; where fine is 2.5 μm or less, PM_2.5_), ozone (O_3_), sulfur dioxide (SO_2_), and oxides of nitrogen (NOx) contribute to a variety of illnesses, including respiratory and cardiovascular diseases, cancer, and stroke (Rojas-Rueda et al., 2021). Substantial variations in ambient air quality and health burdens exist globally, with nearly the entire population (99%) of the Middle East and North Africa facing PM exposures exceeding World Health Organization (WHO) guidelines (Abbass et al., 2018). Saudi Arabia faces some of the greatest burdens (Rojas-Rueda et al., 2021; WHO, 2016, WHO, 2021; these stem from a mix of anthropogenic sources (e.g., transportation, industrial activity, urbanization) and environmental sources such as desert sand (Omidvarborna et al., 2018). As a result, Saudi Arabia experiences deaths attributable to outdoor air pollution at a much greater rate than other countries of comparable income (Our World in Data, 2019; World Bank, 2020).

The production of industrial chemicals, petroleum refining, and the combustion of fossil fuels are leading anthropogenic sources of NOx, SO_2_, and PM in Saudi Arabia (Omidvarborna et al., 2018). Common sources of combustion emissions in Saudi Arabia are transportation, electricity, and water desalination. Transportation emissions include ground transportation in addition to tanker and container ships, whose high-sulfur fuels contribute to SO_2_ pollution. Saudi Arabian shipping supports large trade volumes of 7.1 million barrels per day of oil and 8.9 million twenty-foot equivalent units (TEUs—i.e., shipping containers) (World Bank, 2021). Oil exports are supported primarily by Saudi Arabia’s ports on the gulf (Ras Tanura, with Saudi Arabia’s largest refining capacity) and the Red Sea (Yanbu King Fahd). Saudi Arabia’s electricity generation is supported by 40% oil-burning capacity, a far larger share than that of other nations with comparable income levels, with natural gas accounting for the great majority of the remaining generation capacity. There is currently only negligible capacity in non-emitting sources of electricity, though nuclear and renewable capacity has been planned (U.S. Energy Information Administration, 2014). Water desalination is a major source of electricity demand, supporting 60% of Saudi Arabia’s water consumption. Sandstorms, particularly in the spring and summer, are the leading natural cause of PM pollution.

In response to the long-standing global public health challenges posed by poor ambient air quality, the WHO provides air quality guidelines for addressing health burdens associated with pollution (WHO, 2005, WHO, 2021). Air quality standards commonly identify ambient concentration thresholds that safeguard public health and trigger actions to reduce emissions and/or provide public communication when breached. These actions address pollution’s health risks by requiring emitters to reduce their pollution output (i.e., mitigation) or by supporting public avoidance of ambient pollution exposure (i.e., adaptation). Countries that implement ambient air quality standards must evaluate how to set standards that are consistent with epidemiological evidence and how to apportion ambient pollution concentrations to its sources, monitor and report emissions, and enforce compliance with standards to achieve mitigation. Adaptation measures are particularly important for environmental pollution whose sources cannot be controlled, such as particulates from desert sand, or pollutants whose sources are challenging to regulate. For example, adaptation might include improving indoor air quality through filtration or supporting public avoidance of outdoor areas in times of poor air quality through the dissemination of air quality alerts.

There exists a substantial body of literature (Lipfert, 2018; Rojas-Rueda et al., 2021) on the benefits of avoided morbidity and mortality associated with reducing air pollution. Considerable effort has been expended by public health and environment authorities globally to evaluate and synthesize the combined evidence from epidemiological, atmospheric, and economic sciences in regulating anthropogenic pollution sources. These efforts frequently incorporate cost-benefit analyses that compare monetized benefits of reduced exposure with the cost of achieving it, a critical element of policy making. Many policy options also exist for incentivizing adaptation that must be carefully evaluated to design efficient and effective interventions that will improve environmental public health while limiting the economic costs of achieving those improvements.

Given the breadth of options and cost-benefit information available to policy makers, this research is designed to assess existing evidence and recommendations on effective options for addressing environmental public health burdens in Saudi Arabia. We conduct a semi-systematic literature review of available costs and effectiveness information to address two research questions relevant for environmental public health interventions:

(1) Adaptation: What effective technologies and policy options are available for avoiding exposure to existing air pollution and what are the key drivers of their effectiveness relevant to the Saudi Arabian context?
(2) Mitigation: What cost effective technology and policy options are available for preventing air pollution and what are the key drivers of their costs relevant to the Saudi Arabian context?

The literature review addressing each question is designed to cover the two distinct approaches to addressing environmental public health concerns by either (1) adapting to existing pollution through avoidance measures or (2) mitigating air pollution sources through pollution control measures. We evaluate the availability, content, and relevance of cost and effectiveness information from our search results for efficiently and effectively addressing the prevailing sources and ambient pollution in Saudi Arabia.

## 2 Material and methods

Our research questions are broad and thematic. They are not concerned with investigating the evidence of a single quantitative relationship (e.g., between a specific pollutant and health outcome). Broad, inter-disciplinary, and thematic research questions such as ours are best addressed by a semi-systematic review of available information (Snyder, 2019). In our case, we are identifying themes and limited quantitative summaries of cost effectiveness across multiple different pollutants whose mitigation and adaptation strategies differ. The perspective gained by this approach is designed to serve policy makers in assessing their options across a range of environmental public health options. In this section, we outline our semi-systematic review approach to identifying literature relevant to the two research questions for air pollutants of greatest public health concern.

**Figure 1:**
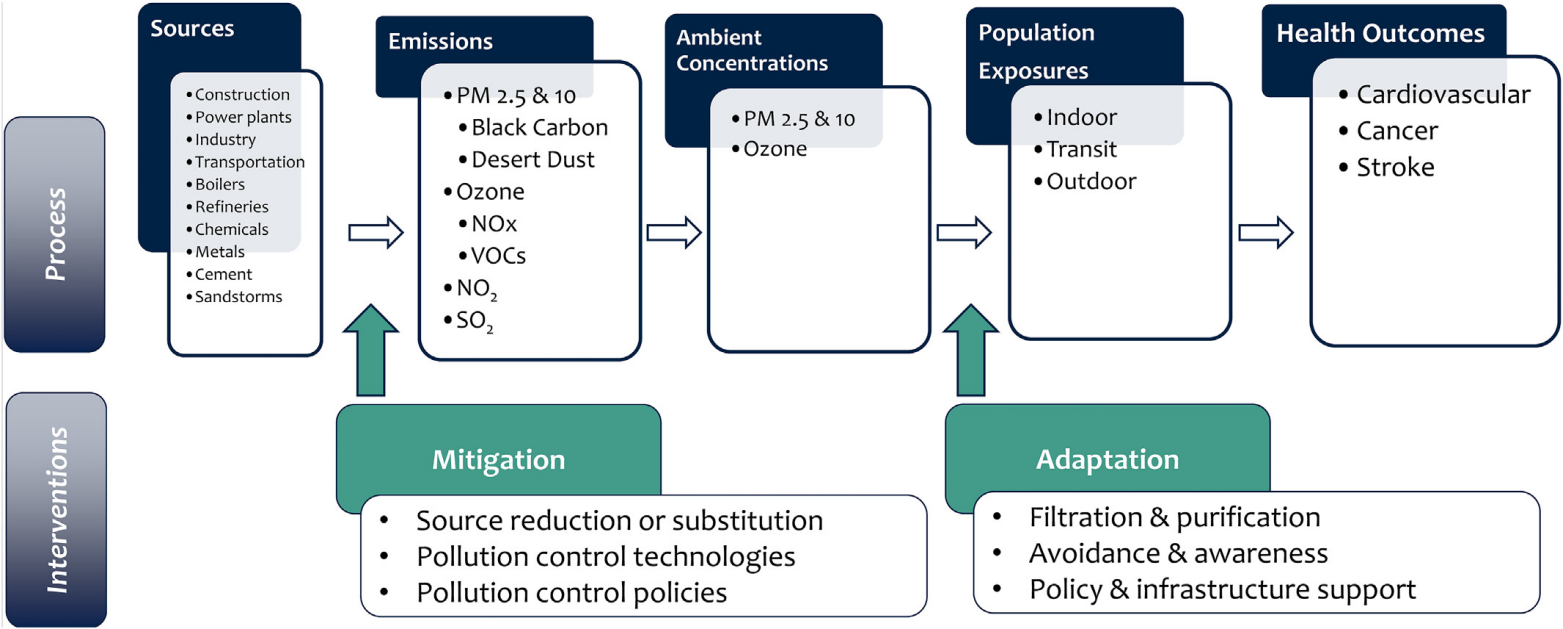
Environmental public health process.

We orient the search strategy on existing public health guidelines from the WHO on ambient air quality (WHO, 2021), which cover four types of pollution: PM, O_3_, NO_2_, and SO_2_. The ambient concentrations of these pollutants are the results of the release, chemical reaction, and movement of a variety of pollutants, a process described as “chemical fate and transport.” For example, there is very little direct emission of O_3_ into the atmosphere, rather it is formed by the interaction of volatile organic compounds (VOCs) and oxides of nitrogen (NOx) in the presence of sunlight. Broadly speaking, to address public health burdens associated with poor air quality, societies must either mitigate emissions or adapt to their presence by avoiding exposure to high ambient concentrations. As these are distinct approaches generally addressed by different bodies of literature, our literature searches are structured to address these two options separately. Figure 1 provides a stylized diagram of the environmental public health process and the points of intervention addressed by our semi-systematic literature review.

### 2.1 Search strategy

For adaptation, we first identified options—ranging from air filtration to air quality warning systems— for avoiding poor ambient air quality. For mitigation, we searched for both technologies and policies designed to reduce air pollutant emissions. Each search includes the pollutants relevant to the air quality hazards identified by the WHO. Literature searches for pollution mitigation (i.e., technology and policy) included pollutants different from those used for adaptation searches because each addressed different ends of the chemical fate and transport process, as noted above. We conducted literature searches for adaptation responses to reduce exposure to pollution in both the Web of Science and PubMed and literature searches for pollution mitigation in the Web of Science only, with all searches over the years 2011–2021.

We targeted our review of strategies to avoid exposure to existing air pollutants (i.e., adaptation) to identify evidence around the cost and effectiveness of these strategies. Much of the literature returned in searches for pollution adaptation included only observational assessments of the correlation between air pollution and health outcomes; there were relatively few evaluating specific policies, interventions, or actions. Cost data were also rare. Our literature search terms for pollution adaptation therefore included known policies, interventions, and actions based on several published reviews of effective actions to reduce exposure to existing air pollution. Including specific terms for known pollution adaptation interventions ensured that we collected evidence related to known, meaningful interventions. Pollution mitigation technologies and policy costs and effectiveness vary by pollutant and source. To return literature with cost and effectiveness estimates with source specificity, our pollution mitigation literature searches included the largest sources of each pollutant’s emissions in an “or” grouping in addition to pollutants, cost, and either technology or policy.

**Figure 2:**
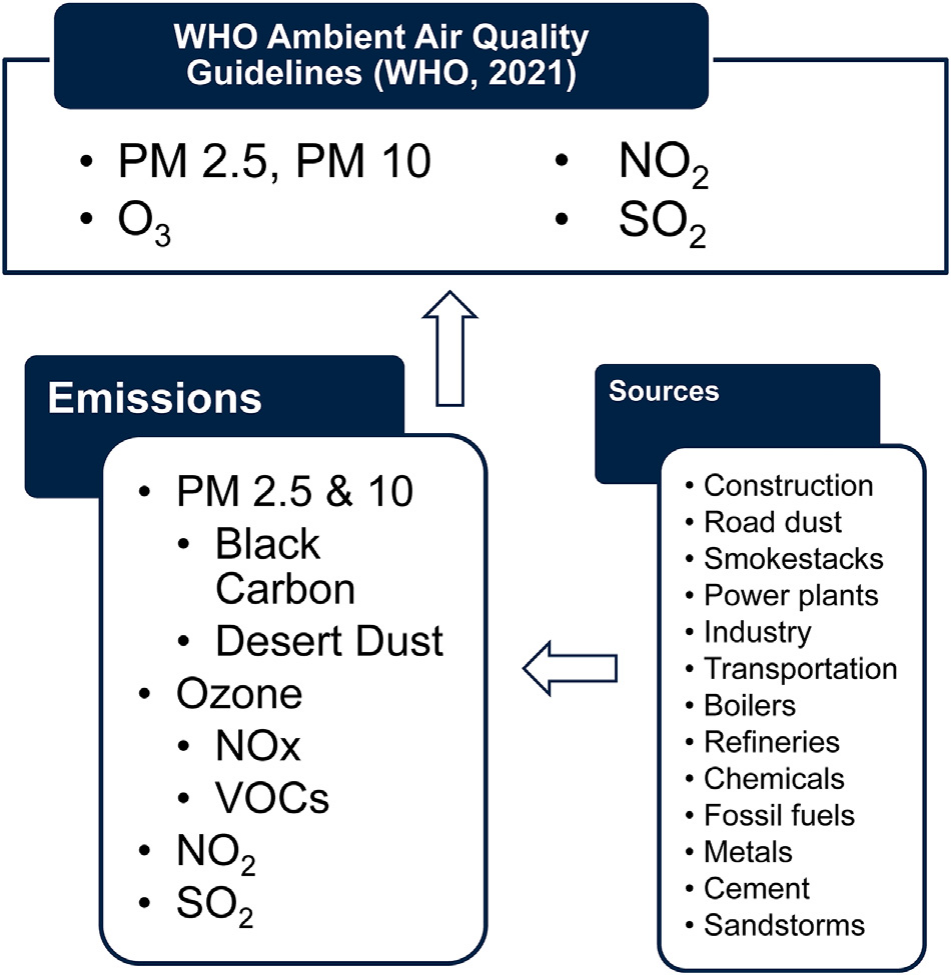
Search term identification.

To identify the costs associated with emissions abatement and pollution control policies, we included precursors to ambient pollution in our searches with their pollutant of concern (e.g., VOCs and O_3_). Figure 2 diagrams the ambient concentrations from WHO guidelines, contributing emissions, and emissions sources that formed the basis of our searches. As pollution control technologies and policies are often specific to the sources of the pollution, we also identified the largest sources of emissions for each pollutant and included all sources in an “OR” group of terms. Appendix A1 provides a complete list of our searches and the terms used for adaptation and mitigation.

In addition to the literature searches described above, we also reviewed and summarized mitigation technology cost information from the US Environmental Protection Agency’s “Menu of Control Measures” for the National Ambient Air Quality Standards, which summarizes control measures and their cost efficiencies for a range of pollutants and sources (US EPA, 2022).

### 2.2 Inclusion criteria

#### 2.2.1 Full article review

We retrieved a list of titles, authors, abstracts, and select other bibliometric information for all our literature search results. We then organized the results by adaptation and mitigation and divided mitigation results into those returned with the search term “policy” and those with the search term “technology,” forming three sets of results for our review. We assigned one author per result set (i.e., adaptation, mitigation technology, and mitigation policy) to review and rate each result on four criteria (enumerated below) based on abstract and title. We removed duplicate results within each of these three sets of results but not across them, leaving results to be scored multiple times and compared to assess inter-rater reliability. We ranked papers as “confident criterion is not met” (0), “not confident whether criterion is met” (0.5), or “confident the criterion is met” (1.0) for each of the four inclusion criteria:

1. Interventions: evaluates specific and relevant interventions such as technologies, policies, or health interventions such that their scope and applicability can be assessed
2. Efficiency
  • Costs: contains quantitative cost or cost-effectiveness information inclusive of ordinal rankings for results from the mitigation search
  • Effectiveness: contains physical effectiveness information for the adaptation search
3. Pollutants: assesses technologies, policies, or health interventions that are relevant to selected pollutants
4. Region: considers technologies, polices, or health interventions that are relevant to public health burdens experienced in Saudi Arabia.

#### 2.2.2 Results scoring at least 3.5 out of 4.0 were included for full text review

**Figure 3:**
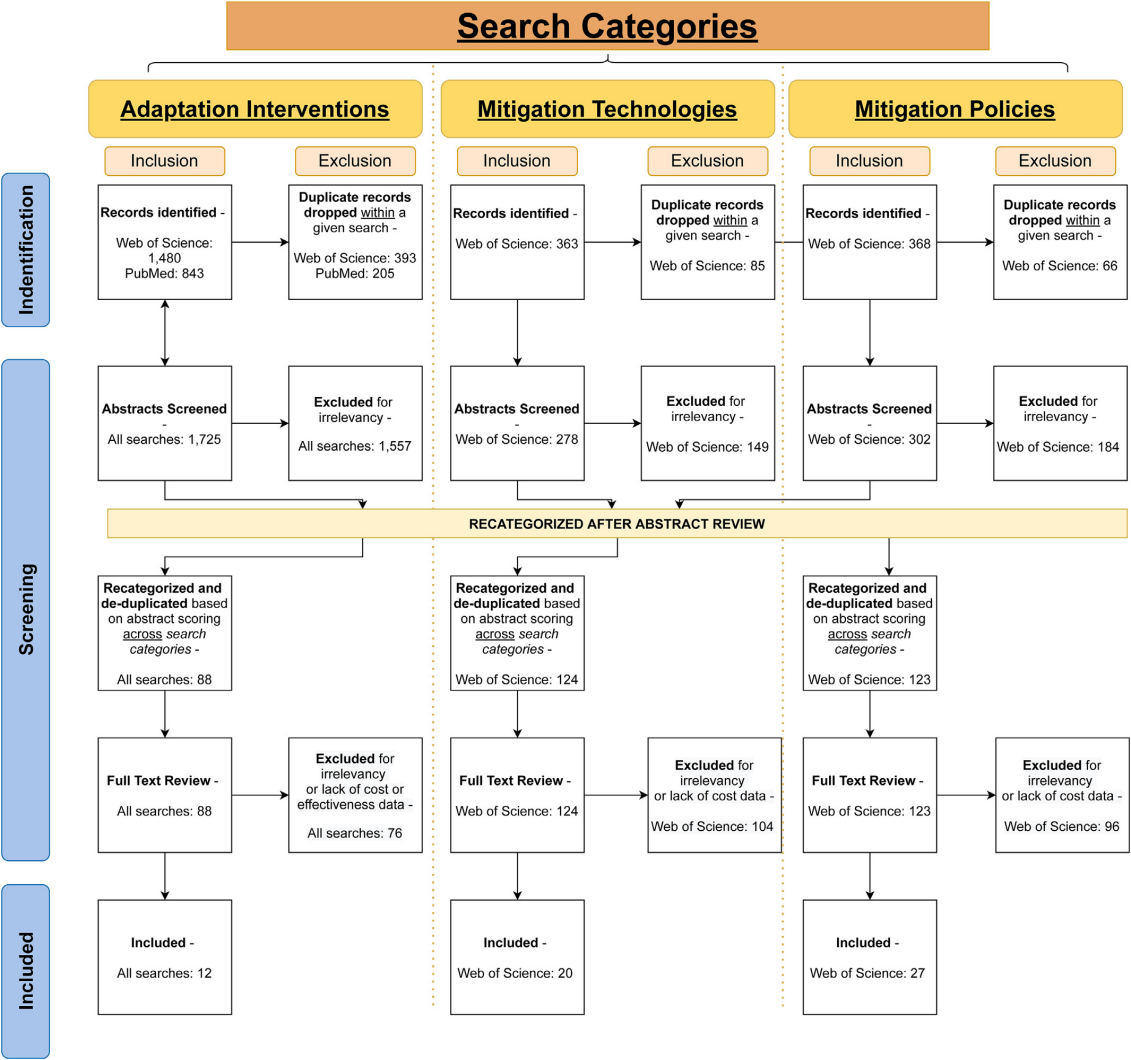
Literature search PRISMA statement.

We marked some results for consideration by other reviewers (e.g., mitigation policy search results that were relevant for mitigation technology). Of the 1,278 abstracts reviewed, 72 were reviewed by more than one reviewer and all received consistent scoring of either 3.5 or greater or less than 3.5. Finally, we re-ranked results in our full-text review on the same criteria as in the abstract review. Figure 3 below enumerates additional details on the inclusion/exclusion of articles by review stage. Data inclusion.

From the full-text reviews, we summarized both qualitative information on mitigation and adaptation interventions and their effectiveness. All papers receiving a score of 3.5 were reviewed for our qualitative summaries. For quantitative mitigation results, we required that the study provide quantitative cost and abatement information in the form of either currency per emission unit reduced (e.g., USD/tonne) or a cost followed by a reduction in emissions so that cost-effectiveness could be calculated. On some occasions, we also had to estimate the pollution reduction amount. Some of the studies provided a baseline value and percent reduction.

Cost values were standardized to common currency-year of 2020 US dollars and emissions values to metric tons. We amortized capital costs from studies reporting large upfront costs using a 30-year timeframe and a 7% discount rate. For cost estimates of mitigation technologies, we extracted costs from the US Environmental Protection Agency’s (EPA) Menu of Control Measures database (US EPA, 2022) to augment the data obtained from the literature search. This database contains estimates of the cost per tonne of emission reductions for a variety of sources including point, non-point, and mobile technologies. The technologies included range from traditional technologies widely used over the past several decades to newer technologies as well. Additionally, the data include several of the key pollutants assessed in this analysis, such as PM, NO_X_, VOC, and SO_2_.

## 3 Results

In total, our literature searches returned 3,068 articles, two-thirds of which came from the adaptation search and 748 of which were duplicates within searches. The mitigation searches returned similar numbers of technology and policy results. The initial screening, based on abstract and title, excluded 1,890 articles that did not receive a scoring of 3.5 or higher in our review. We recategorized articles that were determined to be a better fit for a category other than the one they were initially identified under (e.g., mitigation policy rather than mitigation technology) and removed duplicate articles that were identified in multiple searches, leaving 335 articles for full text review, 276 of which we excluded for not meeting the screening criterion after full text review. The qualitative and quantitative results that follow are based on a full text review of 59 articles passing all stages of our screening. Figure 3 provides a Preferred Reporting Items for Systematic Reviews and Meta-Analyses (PRISMA) statement of our literature review process across our searches. The results reported in Section 3.1 inform research question (1) on the effectiveness of pollution adaptation measures. The results reported in Section 3.2 inform research question (2) on the cost drivers and effectiveness of pollution control technologies. Last, the results reported in Section 3.3 inform research question (2) on the cost drivers and effectiveness of pollution control policies.

### 3.1 Adaptation

The review of adaptation measures to address air quality identified 12 studies that detail effectiveness and/or cost information. Of the studies identified in the review, all discussed effectiveness information and only one discussed cost. The measures identified can be taken by society, a household, and/or an individual. Of those identified, most studies examined air quality messaging systems that use constructed indices for communication to the public. Other studies identified examined measures to reduce human exposure to automobile traffic in urban settings, indoor air filtration and ventilation, and personal mask usage.

Two studies examining effectiveness for ventilation found that improved ventilation significantly reduced human exposure to PM_2.5_. Hong (2019) found that when high-efficiency ventilation was used in light-rail transit, personal exposure to PM_2.5_ reduced by 38% and to black carbon by 68% when compared with car travel at high-traffic times. Tong et al. (2020) compared air filtration units in primary school classrooms in China and found that using filtration units alone (internal recirculation) reduced PM_2.5_ significantly (∼70% reduction), yet it increased carbon dioxide (CO_2_) concentrations sixfold. Ventilating with external air was optimal, as it still reduced PM_2.5_ and mitigated significant CO_2_ build-up. Managing indoor air quality requires both limiting CO_2_ build-up by using outdoor air while also considering relative PM concentrations and air filtration efficiency for outdoor air.

Studies found that indoor air filtration technologies in residential settings were effective in reducing exposure to PM_2.5_, O_3_, and NO_2_, although the context for its usage remains important. Aldred et al. (2016) found that indoor O_3_ removal using commercially available in-duct carbon filters resulted in an increase of ∼5 disability-adjusted life years (DALYs) in high heating, ventilation, and air conditioning (HVAC) usage environments. Fixed costs for in-duct filters make their use cost-beneficial only in settings with heavy HVAC use. Zhan et al. (2018) found that using indoor filters can yield a reduction of more than 40 μg per cubic meter (μg/m^3^) in PM_2.5_ in high air pollution settings (i.e., Beijing households). Paulin et al. (2014) identified an immediate 27% reduction in NO_2_ exposure in urban US residences using unvented gas cooking appliances from the use of air purifiers with high-efficiency particulate air (HEPA) or carbon filters.

Four studies evaluating air quality messaging systems via constructed indices and alert platforms detected at least some positive relationship between the systems on health outcomes for specific groups or for specific diseases. Mason et al. (2019, 2020) identified a correlation between emergency admissions for children (respiratory diseases) and people over 75 (cardiovascular) when China’s air quality index increased, suggesting that, while effective, health indices must ensure widespread adoption. Alari et al. (2021) found that, when Paris’ air quality alert system introduced new, more stringent thresholds for intervention (established at PM_2.5_ = 50 μg/m^3^), a significant impact on cardiovascular disease mortality occurred. Hahm and Yoon (2021) compared two air quality warning systems in South Korea, which disseminate mobile warnings differently; the air quality messaging system that broadcasts warnings to all mobile users (i.e., users need not register to receive notifications) in a specific area reduced patient reporting respiratory disease symptoms by 16.4%, compared with 2.8% for the system that asks users to register to receive notifications, suggesting higher efficacy for opt-out defaults in notification systems.

Three studies identified in our search examined air pollution from automobile traffic and the effectiveness of measures to reduce exposure. Jarjour et al. (2013) examined bicycle commuting routes and found that dedicated bicycle path networks away from high-traffic streets allows bicycle commuters to significantly reduce exposure to ultrafine PM and black carbon. Patel et al. (2016) examined differences in PM_2.5_ exposure among motorcycle and automobile users in Sulawesi, Indonesia, and found that commuting by motorcycle increased PM_2.5_ exposure 4 times and PM_10_ exposure 13 times higher than cars. Gilliland et al. (2019) assessed PM_2.5_ exposure among schoolchildren’s mode of transport to school and found that children who walk instead of taking the bus from home to school experience significantly less exposure.

One study examined the effectiveness of masks in reducing exposure. Patel et al. (2016) evaluated the effectiveness of mask wearing and mask type in relationship to personal PM_2.5_ and PM_10_ exposure in Sulawesi, Indonesia. Surgical masks were found to consistently lower PM_2.5_ (30% reduction) and PM_10_ (71% reduction) exposure, in comparison to no mask, bandanas (26% reduction in PM_10_, average increase in PM_2.5_ exposure), and neoprene motorcycle masks (44% reduction in PM_10_, 2% increase in PM_2.5_ exposure), suggesting a potentially cost-effective role for public health initiatives that subsidize masks and promote their use.

The pollution adaptation literature reviewed offers evidence of effectiveness for five air pollution adaptation options. Table 1 provides a summary of key findings from the review. Building and transit ventilation and filtration systems can reduce exposure between 38% and 70%, depending on the setting, operation, and specific pollutant. Outdoor air, while a key source of air pollution, also helps prevent CO_2_ build-up. Building air filtration such as in-duct HEPA or carbon filters can improve health outcomes by reducing exposure to particulates and NO_2_ (e.g., from unvented gas cooking) provided they are operated regularly. Air quality hazard information can lead to reductions in reported symptoms and hospital admissions of approximately 15%; hazard information is significantly more effective when users are automatically enrolled. Exposure during commuting is substantially higher for motorcycle and bicycle traffic, but separating pedestrians and bicyclists from vehicular traffic and using face masks can significantly reduce exposures.

**Table 1:** Effectiveness of adaptation interventions.

Intervention	Evidence of Reduced Exposure
Face masks *1 study*	PM_2.5_ (−30% PM_2.5,_ −71% PM_10_) Surgical mask during motorcycle commute vs. no mask Sulawesi, Indonesia (Patel et al., 2016)
Ventilation *2 studies*	PM_2.5_ (−38%); Black carbon (−68%) High-efficiency ventilation, public transit vs. autos Los Angeles, USA (Hong, 2019)
	PM_2.5_ (−70%) Air filtration with natural ventilation, primary school classrooms China (Z. Tong, Li, Westerdahl and Freeman, 2020)
Filters *3 studies*	∼5 DALYs avoided; cost: 10 USD In-duct carbon filters used in HVAC in heavy HVAC usage climates United States (Aldred et al., 2016)
	NO_2_ (−27%) HEPA or carbon filter air purifiers, HHs using unvented gas appliances Baltimore, USA (Paulin et al., 2014)
	PM_2.5_ (−40 μg/m^3^) Indoor filters Beijing, China (W. Zhang, Li, Xu and Liu, 2018)
Air quality alerts/indices *4 studies*	Respiratory disease symptoms reported (−16.4%) Alert system, broadcasted to all users in area (auto-enroll) South Korea (Hahm and Yoon, 2021)
	Hospital admissions for respiratory tract infections (−16%) and pneumonia (−12%) Air Quality Health Index alerts Hong Kong, China (Mason et al., 2020; Mason et al., 2019)
	Significant reduction in cardiovascular disease–related mortality Stringent thresholds vs. relaxed thresholds used in air quality warnings Paris, France (Alari et al., 2021)
Traffic separation/transport mode *3 studies*	PM_2.5_ (4 × reduction); PM_10_ (13x reduction) Car versus motorcycle commute Sulawesi, Indonesia (Patel et al., 2016)
	Black carbon (−0.37 μg/m^3^); carbon monoxide (−0.16 ppm) Bicycle commute, routes w/low auto traffic exposure Berkeley, USA (Jarjour et al., 2013)
	Significant decrease in PM_2.5_ exposure Children’s school commute by bus vs. walking Canada (Gilliland et al., 2019)

### 3.2 Mitigation technology

Although there is substantial interest in identifying and characterizing cost-effective technologies for mitigating air pollution, our search of the literature found relatively few studies in the academic literature that meet our search criteria. Generally, there is wide variation in estimated costs associated with alternative mitigation technologies, partly because the cost of reducing a given level of emissions depends heavily on the incremental costs of the mitigation technologies as well as the emissions reduction efficiency available at each emitting facility. Table 2 summarizes the findings of our review of recent literature with sufficient information to derive estimates of cost-effectiveness. We separated the available observations into those based on controlling single pollutants and those assessing the simultaneous control of multiple pollutants. Among the estimates assessing single pollutants, the majority (24 estimates) focused on PM, followed by NO_x_ (11), and SO_2_ (10). The median costs per tonne reduced were similar for PM and NO_x,_ with both exceeding the median cost-effectiveness of mitigating SO_2_ by more than three times. The ranges are so large that they all overlap, though the interquartile range of cost-effectiveness for SO_2_ is substantially lower and narrower than it is for PM or NO_x_. Negative costs imply that some opportunities exist for cost savings (e.g., through efficiency improvements) in controlling emissions.

**Table 2:** Mitigation technology costs.

	2020 USD/Tonne Reduced					
Mitigation Target	Observations	Minimum	Median	Maximum	25th Percentile	75th Percentile
*Single Pollutant*						
PM	24	−3,133	720	104,126	−2,370	11,530
SO_2_	10	2	190	19,125	41	324
NO_X_	11	0.3957	694	11,826	321	5,454
*Multipollutant*						
NO_X_, PM	12	0.82	481	5,417	102	1,075
NO_X_, SO_2_	16	4	115	10,986	4	388
PM, SO_2_	2	204	2,705	5,206	1,454	3,956

*Sources: Authors’ compilation of values reported in literature* (Ammar and Seddiek, 2017; T. L. Chen et al., 2019; Evans, Rojas-Bracho, Hammitt and Dockery, 2021; Galvis et al., 1995; H. Li, Tan, Guo, Zhu and Huang, 2019; F. Liu et al., 2013; Mardones and Saavedra, 2016; Miranda et al., 2016; Nazar et al., 2021; Obara and Li, 2020; Ravina et al., 2020; Shawhan and Picciano, 2019; J. Sun, Schreifels, Wang, Fu and Wang, 2014; F. Tong, Hendrickson, Biehler, Jaramillo and Seki, 2017; Wadud and Khan, 2013; S. Zhang, Worrell, Crijns-Graus, Wagner and Cofala, 2014).

Within the literature reviewed, mitigation technologies assessed include an array of strategies applied to emission sources; these sources include power generation, transportation, manufacturing, industrial, and residential sectors. The recent literature that met our search criteria is heavily focused on transportation relative to other sources, including studies examining on-road passenger vehicles, locomotives, ships, transit buses, and heavy-duty diesel vehicles.

Kanada et al. (2013) explored the potential for cost-effective SO_2_ mitigation in five mega-cities in China, focusing on the implementation of two technologies: flue gas desulfurization and limestone injection. The authors examined potential mitigation and cost-effectiveness using these technologies in the power generation and industrial sectors for each of those cities. Overall cost-effectiveness was estimated to be 19,125 USD per tonne SO_2_, although there was variation across sources and cities. Tong, Hendrickson, Biehler, Jaramillo, and Seki (2017) assessed several different alternative fuel options for transit buses in Pittsburgh, Pennsylvania, including the substitution of traditional diesel fuel with a biodiesel blend, a diesel hybrid-electric bus, compressed natural gas (CNG), liquified natural gas (LNG), and a battery electric bus. They found that all alternative fuel options raise ownership costs but can substantially reduce PM (by 67%–89%) and NO_x_ emissions (by 71%–100%) at costs ranging from 26,521 USD to 45,783 USD per tonne for PM and 433 USD to 858 USD per tonne for NO_x_. Evans, Rojas-Bracho, Hammitt and Dockery (2021) assessed the benefits of retrofitting of heavy-duty diesel vehicles in Mexico City and estimated expected annual health benefits of 250 million USD associated with reductions in PM_2.5_ achieved at a cost of 92 million USD per year in their primary case. Cost-effectiveness in their scenarios ranged from 14,973 USD to 104,126 USD per tonne of PM reduced.

Estimation of the costs associated with regulations designed to reduce air pollution is an important part of the regulatory process in many middle- and high-income countries that are implementing air quality regulations. Typically, detailed engineering analyses are conducted on behalf of regulatory agencies to assess the current state of technology in industries that may be subject to regulation as well as the costs associated with requiring additional controls at facilities operating within those industries. These studies are typically published as independent technical reports and their technical results can remain valid for long periods of time (e.g., decades), especially in mature industries that rely on emissions-generating technologies with only modest technical change. Inasmuch as the maturity of the emissions-generating technologies suggest global diffusion of these technologies (e.g., fossil fuel combustion, industrial processes), the maturity of applicable emissions control technologies means that they are likely to be transferable to uncontrolled sources in other locations such as Saudi Arabia. However, performance characteristics may vary significantly with environmental conditions, thus leading to different cost effectiveness.

That pollution control cost estimates tend to be published in technical reports and are valid for many years is consistent with our finding relatively few studies with data on the cost-effectiveness of alternative mitigation technologies in the recent academic literature. To address this literature gap, we supplemented our review of the academic literature with information available from the US EPA pollution control measures database (US EPA, 2021). The US EPA has been incorporating information into this database for many years, adding new data as it has been developed for regulatory purposes.

Control cost data can be used to inform an assessment of the potential costs of achieving mitigation goals across affected industries. As shown in Table 3, there is wide variation in the estimated cost-effectiveness of mitigating major air pollutants, reflecting differences across industries, technologies, and individual types of facilities within affected industries. Ranges are presented separately for mobile sources versus point and non-point sources. Overall, the cost range for mitigating NO_x_ tends to be higher for mobile sources, while costs for mitigating VOCs and PM are higher for point and non-point sources.

**Table 3:** EPA control measure costs (2020 USD per tonne).

	NO_X_			VOCs			PM			SO_2_		
	Low	Median	High	Low	Median	High	Low	Median	High	Low	Median	High
Point & Non-Point	0	10,557	21,114	−2,345	21,539	45,424	38	48,085	96,132	226	40,444	80,662
Mobile Sources	0	44,461	88,922	0	4,033	8,066	0	12,736	25,472	n.a.	.n.a.	n.a.

*Source:*US EPA (2022).

*Note:* NOx = oxides of nitrogen; PM = particulate matter; SO_2_ = sulfur dioxide; VOCs = volatile organic compounds.

Although costs per tonne of abatement can reach thousands to tens of thousands of dollars, benefit estimates for these pollutants are also quite large. The US EPA estimates PM_2.5_ and O_3_-related benefits of 56,604 USD to 125,166 USD per tonne reduced for NO_X_, 1,783 USD to 35,541 USD for VOCs, 67,278 USD to 515,880 USD per tonne for directly emitted PM_2.5_, and 10,912 USD to 78,095 USD per tonne for SO_2_ (US EPA, 2021, Table 9, converted to 2020 US dollars per tonne), indicating economically efficient abatement opportunities (i.e., mitigation options for which abatement benefits are greater than costs). Variation in these values reflects differences in the estimated benefits across the 21 sectors analyzed, though the primary underlying driver of variation is comprised of the differences in the number of people impacted by reductions in these pollutants in each sector across the United States.

A given quantity of pollution reduction provides a larger monetized benefit in more densely populated areas. Thus, differences in benefits provided by different sectors relate to the geographic locations of their emissions. Data on SO_2_ mitigation costs from mobile sources are not provided in EPA pollution source data because coal combustion is the primary source of SO_2_ emissions and is not used at an appreciable scale in transportation. Certain petroleum products (e.g., diesel or bunker fuels, which are common in shipping) emit SO_2_, but these sources are either negligible relative to total emissions or, in the case of international shipping, are not controlled by domestic regulatory bodies. Indeed, policy options for controlling shipping emissions is a rich area of recent policy study (see Section 3.3).

**Figure 4:**
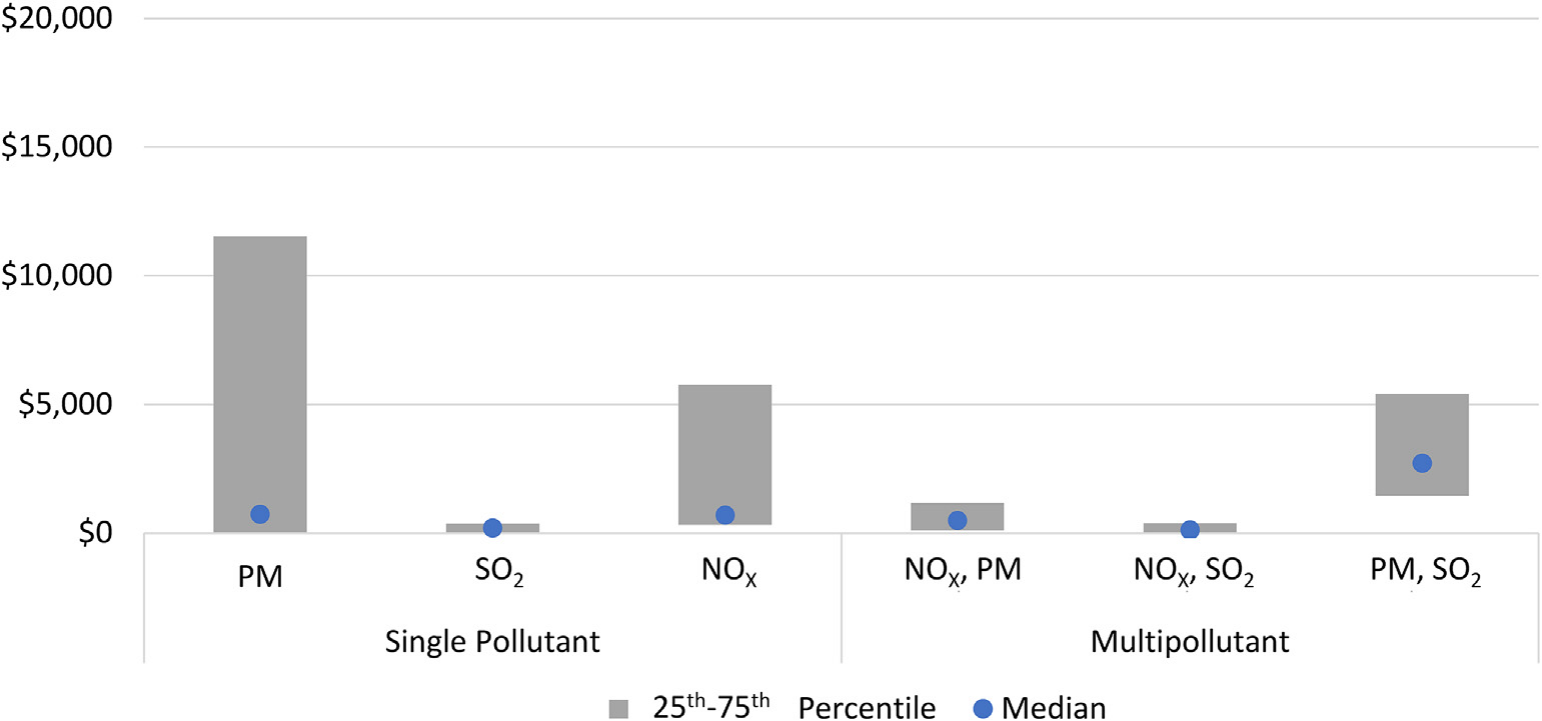
Technology Costs (USD/ton) by Pollutant from Literature; *Sources: Authors’ compilation of values reported in literature* (Ammar and Seddiek, 2017; T. L. Chen et al., 2019; Evans et al., 2021; Galvis et al., 1995; H.H. Li et al., 2019; F. Liu et al., 2013; Mardones and Saavedra, 2016; Miranda et al., 2016; Nazar et al., 2021; Obara and Li, 2020; Ravina et al., 2020; Shawhan and Picciano, 2019; J. Sun et al., 2014; F. Tong et al., 2017; Wadud and Khan, 2013; S. Zhang et al., 2014).

Theoretically, multipollutant control strategies can reduce the costs of achieving given levels of pollutant reduction relative to strategies focused on individual pollutants. Many pollution sources emit multiple pollutants and certain control technologies reduce emissions of multiple pollutants simultaneously (e.g., SO_2_ scrubbers can also remove PM and other pollutants; fuel switching may affect emissions of multiple air pollutants as well as greenhouse gases). In addition, lower emissions for precursor pollutants such as NO_x_ can contribute to reductions in both PM and O_3_ concentrations. Thus, developing integrated strategies designed to address multiple pollutants at once as opposed to applying independent strategies for each has the potential to increase efficiency. In Figure 4, we observe that the average costs per tonne reduced reported for the multipollutant combinations of NO_x_ and PM as well as for NO_x_ and SO_2_ are lower than for these pollutants individually and within smaller ranges. This is not the case for PM and SO_2_, however, though there were only two observations that explored this combination of multipollutant reductions. It is important to keep in mind that these values are indicative of the values presented in the literature but are not directly comparable because they reflect different sets of mitigation options across different studies.

**Figure 5:**
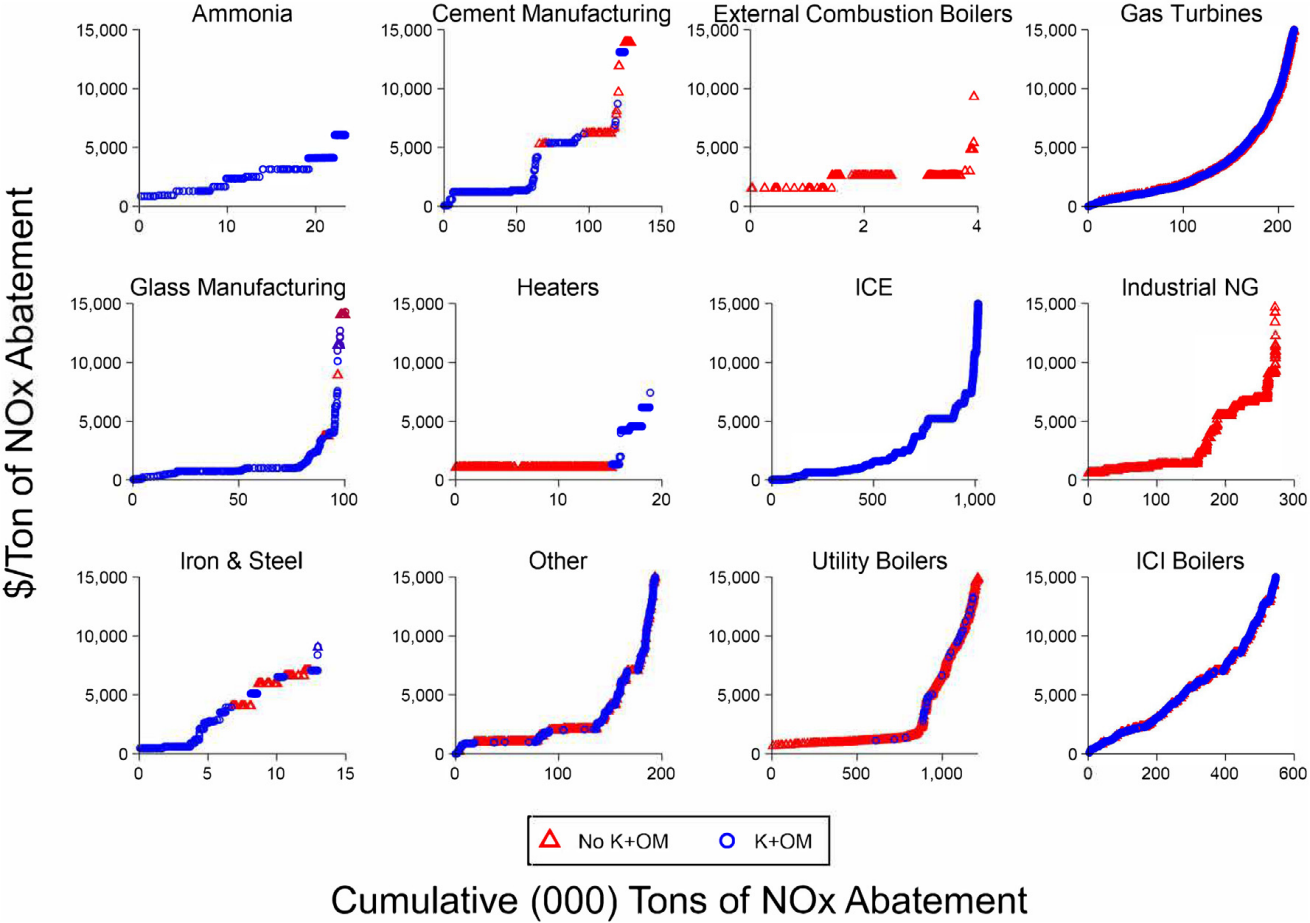
Marginal Abatement Cost of NO_X_ by Sector and Cost Data Availability. *Note*: K + OM indicates observations that also include capital and operations and maintenance cost information. ICE = internal combustion engine; NG = natural gas; ICI = industrial, commercial, and institutional. Ranges for horizontal axes vary. Conversion to metric tonnes is USD/ton ∗ 1.1023 = USD/tonne; *Source*: Click or tap here to enter text (RTI International, 2020, Figure 2).

As noted above, there are many mitigation opportunities available at costs less than the value of associated health benefits. However, given the large variation in costs not only across sectors but also within sectors, it is very important to disaggregate the estimated costs to identify where cost-effective emissions reduction opportunities are available. Figure 5 presents marginal abatement cost curves (MACCs) estimated for NO_x_ reduction across 12 US sectors (RTI International, 2020). MACCs represent the mitigation potential available at different costs, ordering mitigation opportunities by cost-effectiveness. Thus, as one moves from left to right along the x-axis of the curves, an increasing amount of mitigation can be achieved, though at rising costs as shown on the y-axis. In Figure 5, while all sectors are shown with the same cost scale on the y-axis, the scale of each x-axis is very different, reflecting large differences in the magnitude of potential reductions available from individual sectors. Potential reductions shown at prices up to about 15,000 USD per tonne of NO_x_ reduction vary from about 4,000 tonnes for external combustion boilers to about 1,250,000 tonnes for utility boilers (note that Figure 5 is presented in short tons or 0.907 tonnes). This difference in mitigation potential is a function of the baseline emissions generated by different sectors, the reduction effectiveness of available mitigation options in each sector, and the cost-effectiveness of those options.

In addition to the differences in technical potential at a given cost, MACCs provide valuable insights into the ways that cost-effectiveness changes as mitigation is increased. The potential level of cost-effective mitigation available from different sources is an important input to policy design. In most sectors, there are very large differences in cost-effectiveness. As more and more mitigation is achieved in a given sector, the marginal cost of additional mitigation tends to reach an inflection point because low-cost mitigation opportunities have already been adopted and further mitigation must be achieved by using very expensive technologies. Instituting policies that incentivize efficient mitigation can greatly reduce the costs of meeting a mitigation target.

### 3.3 Mitigation policies

Mitigation policies are designed to encourage market participants to put into effect pollution control technologies that would not be privately economical. Pollution mitigation policies are innumerable in their specific provisions and regulatory requirements. In terms of categories, they include one or more of the following mechanisms to induce the adoption of pollution controls: (1) taxes or subsidies provide direct economic penalties or incentives for polluting for mitigating pollution; (2) permitting schemes create a restricted commodity for pollution, limiting a quantity or emissions rate; (3) “command-and-control” approaches require specific action by emitters; (4) public information and other non-pecuniary and non-remunerative policies can help encourage the adoption of mitigation options where individuals or firms may find it privately beneficial to do so given sufficient information (e.g., explaining the cost savings of energy efficiency upgrades).

There exist many pollution control technologies, many policy options for pollution control, many pollution sources from which to control, and many different economic and policy settings in which to implement environmental policies. This diversity of conditions for pollution control is evident in the wide variability in pollution control policy costs. Table 4 shows considerable variation across pollutants in policy costs as calculated from our reviewed literature. Pollution control costs for SO_2_ and NO_X_ are significantly higher than technology costs, suggesting the amount of abatement per technology and broader economic impacts may be quite important areas of study. Conversely, PM control policy costs are significantly lower than their technology costs, suggesting the most expensive mitigation technologies may not be needed to reach common policy targets. The large variation in policy costs underscores the importance of understanding sector and pollutant specific mitigation cost curves to evaluate the cost effectiveness of different policy options.

**Table 4:** Mitigation policy costs from literature review.

	2020 USD/Tonne reduced					
	Obs.	Minimum	Median	Maximum	25^th^ Percentile	75^th^ Percentile
*Single Pollutant*						
PM	**16**	11	167	71,872	34	6,727
SO_2_	**39**	2	780	68,840	627	2,792
NO_X_	**61**	0	1,550	146,739	354	4,552
*Multipollutant*						
NO_2_, SO_2_	**7**	234	3,773	15,688	3,245	13,338
PM, NO_X_	**3**	-901	4,252	10,581	1,676	7,417
PM, NO_X_, SO_2_	**8**	77	358	2,731	186	1,186
PM, NO_X_, SO_2_, VOCs	**1**	1,139	1,139	1,139	1,139	1,139
PM, NO_X_, SO_X_	**4**	4	9,051	115,433	17	42,419
PM, NO_X_, SO_X_, VOCs	**2**	2,058	2,203	2,349	2,131	2,276
PM, NO_X_, VOCs	**2**	15	3,364	6,713	1,690	5,039
PM, NO_X_, HC, CO	**2**	791	1,704	2,617	1,248	2,160
PM, SO_2_	**24**	0.31	8	286	2	34

*Sources: Authors’ compilation of values from literature* (F. Chen, Yamashita, Kurokawa and Klimont, 2015; Chiesa et al., 2014; Fowlie et al., 2012; Guo et al., 2018; Hasanbeigi et al., 2013; Howard et al., 2019; Lai et al., 2020; N.N. Li et al., 2019; H. Liu, Meng, et al., 2018; Mardones and Cabello, 2019; McDonald-Buller et al., 2016; Miranda et al., 2016; Peng et al., 2019; Pinchasik et al., 2020; Raff and Walter, 2019; Relvas and Miranda, 2018; Rodgers et al., 2019; Sanderson et al., 2013; Shih and Tseng, 2014; L. Sun et al., 2012; Taksibi et al., 2020; K. Wang et al., 2020; L. Wang et al., 2016; Sheng Wang, Qing, Wang and Li, 2018; Shijie Wang et al., 2019; Xiao et al., 2019; Xu et al., 2021; Zhou et al., 2019)*.*

*Note:* CO = carbon monoxide; NOx = oxides of nitrogen; PM = particulate matter; SO_2_ = sulfur dioxide; VOCs = volatile organic compounds; HC = hydrocarbons.

There exists broad support in the literature for the cost effectiveness of policies with greater flexibility in how pollution abatement is achieved. By contrast, command-and-control policies have generally been found to be among the least cost effective options for pollution control, though output restrictions can be effective strategies for the “emergency control of heavy pollution,” particularly where sources are limited and excess capacity mitigates economic impacts for consumers (Xu et al., 2021). Cap-and-trade policies have been found to be far more cost efficient than policies that subsidize clean alternatives (Saari et al., 2014; Thompson et al., 2014), though the two may be synergistic and cost-effective when deployed together (L. Wang et al., 2016). Indeed, recent empirical estimates for Iran, another major oil-producing state in the region, suggests that consumers may exhibit significant demand response to policies that raise the price of fossil fuels, which could offer significant air quality improvements (Khatibi et al., 2020). Shih and Tseng (2014) found that energy efficiency policies may be less efficient still than policies that subsidize clean alternatives, in part because of rebound effects where efficiency cost savings lead to higher demand. Residential pollution sources are a key source for unrealized efficiency gains (Chiesa et al., 2014), where there exist many mitigation options that can produce cost savings, suggesting a role for information campaigns, appliance financing support for homeowners, and policies that help resolve poor incentive structures in rental residences.

Pollution emissions are jointly produced, so any policy targeting one pollutant is likely to have spillover effects on others. The cost-effectiveness of policies directly targeting pollutants tends to be much higher than policies that reduce those pollutants as a co-benefit of other policies (Pinchasik et al., 2020), and policy co-benefits may be diminished when other pollutants are already controlled (Mardones and Cabello, 2019). That said, policy co-benefits, particularly from greenhouse gas policies, can be significant (Schucht et al., 2015; Woollacott, 2018). Examining economy-wide tax-based policies in the Chinese economy to reduce SO_2_, NO_X_, and soot and dust emissions (K. Wang et al., 2020), considered independent and joint taxes across the different pollutants and found the greatest policy benefits from soot and dust taxation. Their independent and joint variation of tax levels is a valuable way to identify efficient outcomes, but studies simulating multipollutant interactions in this way were rare in our search results and their effective implementation requires rich engineering and economic characterizations of pollution-generating processes and their controls.

Policies that affect the greatest reductions in pollution are not necessarily the most economically efficient (K. Wang et al., 2020); rather, identifying efficient outcomes requires a careful consideration of a policy’s marginal costs and benefits where the optimal price of emissions damages (i.e., abatement benefit) or the quantity of emissions abatement is probably not known with certainty. Moreover, considerable temporal and spatial heterogeneity in benefits and costs complicate policy design further. For example, pollutants released during daylight hours with full sun will face a different fate than the same emissions on a cold night. This temporal heterogeneity in emissions damages can lead to significant inefficiencies for polices that are not time differentiated (McDonald-Buller et al., 2016). Certain operational strategies (e.g., the timing of when certain technologies are dispatched on the electric grid) can deliver some of the potential efficiency gains comparable to the value of installing new control technologies, but these strategies are subject to the availability of accurate forecasts (Sun et al., 2012). Particulate matter is a prime example of spatial heterogeneity, as its concentrations can vary significantly over relatively fine spatial scales (Cheng et al., 2019), leading to significant demographic differences in exposures.

Broad air quality guidance such as that promulgated by the WHO provide a valuable reference point for policy making, but significant heterogeneity in costs and benefits from one location to another may render such guidance inadequate for locally optimal policy design. Indeed, some have found economically efficient abatement opportunities may exist well beyond WHO guidelines (Howard et al., 2019). In evaluating local environmental, policy, and economic conditions, policy makers must balance the cost-effectiveness of broader, more flexible policies with the value of addressing the spatio-temporal heterogeneity in abatement costs and benefits.

The literature we reviewed suggests that the cost efficiency of cap-and-trade policies can be enhanced by broadening policies to allow for emissions trading among different control regions. However, a key drawback of this approach is the potential for inequitable distribution of costs and benefits across individuals, regions, and industries. Even if trading rendered total benefits equal and costs lower, the distributional equity of policy costs and benefits remains an important concern for policy makers to weigh against potential efficiency gains. Pollution-intensive regions may face both high incidence policy costs and high abatement benefits, raising the stakes for effectively making efficiency-equity tradeoffs in policy design. Zhang, Wu, and Choi (2020) emphasized the need for regional policy differentiation—for example, by establishing exchange rates for permits traded across control areas that can help mitigate such inequitable benefit distributions (Peng et al., 2019). Transfer payments can also help manage equity-efficiency tradeoffs where they are politically viable —for example, where pollution travels over political boundaries (Dong et al., 2015).

Emissions control policies are often limited in spatial extent, either for jurisdictional reasons or to address particularly poor air quality in a certain area. Jurisdictional issues are particularly problematic for where pollution travels across boundaries (Taksibi et al., 2020). For example, Saudi Arabia may be exposed to pollution from North Africa that it cannot control. Policy simulations that provide detailed atmospheric modeling (Saari et al., 2014) can help assess spatial and temporal heterogeneity in benefits, but such modeling must incorporate the timing and quantity of all relevant emissions and all their sources, natural and anthropogenic; meteorological simulations calibrated to historical conditions; and calibrated stoichiometric relationships among pollutants and their precursors. Spatial downscaling techniques in areas with dense and heterogeneous populations may also be required, all adding to the complexity and resource requirements of policy designs that account for fine spatio-temporal variability.

Poor air quality can be particularly problematic in heavily industrialized or dense urban areas with high traffic densities. The literature we reviewed contained many studies focused on transportation, where spatial clustering of sources (e.g., traffic) leads to recurrent challenges with local pollution. Low emissions zones for road traffic have been found to be cost-effective at reducing PM and NO_X_ (Rohlf et al., 2020). Studies of early retirement programs for vehicles found that subsidizing the retirement of the least-efficient, often oldest vehicles was more efficient than restricting the total volume of travel (Xiao et al., 2019), and the cost-benefit ratios of subsidizing the retirement of relatively dirty, “yellow label” vehicles in China has been found to be greater than 1 (Zhou et al., 2019). Second-hand markets may diminish the benefits of subsidized retirements, however. In the United States, the Consumer Assistance to Recycle and Save Act of 2009 (CARS, also known as the ‘Cash for Clunkers’ program) addressed leakage by scrapping retired vehicles; similar programs have been implemented in other Organisation for Economic Co-operation and Development (OECD) countries (CRS, 2020).

In maritime travel, international container ship traffic is a major contributor to poor air quality around ports, but container ships are subject to local jurisdiction only when near a port. Once in international waters, they are subject to regulations agreed through the International Maritime Organization. Emissions control areas (ECAs), which impose certain idling restrictions or require the combustion of low-sulfur fuels, are a common policy tool for addressing port pollution, but evidence on their effectiveness is mixed. For example, Antturi et al. (2016) found that an ECA established in the Baltic Sea did not pass a cost-benefit test, though the authors did not consider all benefits. ECAs may not cover all relevant emissions for certain ports. For example, high container ship traffic outside ECAs may still contribute to pollution around ports under certain prevailing meteorological conditions. Liu et al., 2018a, Liu et al., 2018b found that regional control measures offer significantly more emissions reduction than port controls.

The broader global freight industry’s function of making point-to-point delivery of commodities and other goods across different geographies and fixed transportation infrastructure faces complex and tightly constrained logistics. Within these networks, Pinchasik et al. (2020) found that modal shifts from road to rail freight can improve efficiency, but freight system logistics may significantly constrain such opportunities. They also found that policy harmonization across countries, effectively expanding the policy region as studied by Liu et al., 2018a, Liu et al., 2018b, can improve policy effectiveness where one locality may not have sufficient economic influence on these networks to effect significant changes in technology or operations.

Not all heterogeneity in pollution sources are relevant drivers of policy costs and benefits. While the literature has shown that spatio-temporal heterogeneity in costs and benefits are important factors in policy design, pollution source types may not be. That is, for example, NO_X_ emissions from a motorcycle tailpipe idling outside a restaurant burning natural gas for cooking (i.e., in the same time and place) should be treated equally, but often are not in practice. Such source-differentiated regulations, arising from political economy or administrative reasons, can lead to significant policy inefficiencies, suggesting a need for greater policy coordination across regulatory programs (Fowlie et al., 2012).

Furthermore, there are practical limits to what can be done to maximize policy efficiency. While economic efficiency can be gained through more spatio-temporally differentiated policies, the monitoring, transactions, and enforcement (Howard et al., 2019) costs of incorporating this information may practically limit the extent of efficiency gains from a temporally differentiated policy. More temporally and/or spatially nuanced policies may offer greater efficiency in theory while at the same time lacking practical policy information, verifiability, and/or enforceability (S. Wang et al., 2018), leaving efficiency gains elusive.

Policy effectiveness also depends on certain and significant price signals to private market participants to induce abatement activity. The long-term temporal distribution of costs and benefits is an important consideration in policy evaluation (Raff and Walter, 2019), as large upfront capital costs for pollution controls must be recouped over years of recurrent public benefits via air quality improvements and private benefits via policy instruments. Benefit projections are uncertain since pollution sources, populations, and climate (Schucht et al., 2015; Taksibi et al., 2020) are all subject to change, which drives different realizations of policy benefits. These longer-term uncertainties challenge policy design and implementation, as private market participants may discount uncertain future benefits from policy instruments such as production tax credits, which pay out over time instead of in the near term as with investment tax credits.

Last, there may exist behavioral limitations to the effectiveness of policies designed with rationality assumptions. Residential mitigation may be more likely to suffer from incomplete or improper use of mitigation technologies, substantially undermining effectiveness in practice and emphasizing the need for policy compliance monitoring, consideration of its practicality and costs, and careful analysis of behavioral factors (Mardones, 2021).

## 4 Discussion

### 4.1 Adaptation

Saudi Arabia faces a mix of pollution mitigation challenges. Natural sources such as desert sand cannot be effectively prevented, there exist anthropogenic sources beyond Saudi Arabia’s control that influence its ambient air quality, and certain pollutant sources may be prohibitively expensive to mitigate effectively. Our review addressed our first research question by first identifying several adaptation options for poor air quality and then evaluating the literature on their effectiveness. Effective adaptation strategies included indoor air filtration, ventilation, traffic separation, public air quality information services, and face masks. The existing literature suggests that all these adaptation options have the potential to significantly reduce pollution exposure to varying degrees.

Our review identified certain key drivers of the effectiveness of these adaptation options. In indoor ventilation and filtration, HVAC design must factor tradeoffs with other aspects of indoor air quality such as O_2_–CO_2_ balances and thermal efficiency. Because HVAC systems are designed primarily to provide thermal comfort, locations and seasons with modest heating and cooling requirements will provide only modest filtration benefits unless operations are modified, though this issue will be less acute for Saudi Arabia, which has persistent cooling needs. Independent air filtration devices are also effective at reducing pollution exposure, and the relative cost effectiveness between those devices and HVAC integration requires further cost-benefit analysis that considers the cost variation across existing and new building equipment.

With indoor air quality improvements, air quality alert systems are likely to be an effective way for Saudi Arabia to encourage public avoidance of pollution exposures, with opt-out programs providing greater efficacy than opt-in programs. Outdoors, civil infrastructure design can significantly reduce exposure for pedestrians and cyclists by separating them from traffic, but cost-effectiveness evaluations of these alternatives are lacking in the literature and are likely to vary significantly by locality, as design costs depend on existing infrastructure and geography. Existing studies can help Saudi officials identify effective separation distances and/or barriers given local emissions intensity and meteorology, but cost evaluations will probably need to be conducted on a project-by-project basis. Last, as a low-cost alternative, and where clean air is a challenge to access, face masks can provide significant exposure reduction.

### 4.2 Mitigation

In addressing our second research question on cost effective mitigation options, our literature review identified that, where pollution emissions can be mitigated, the costs of doing so vary widely. This variance in costs potentially limits the generalizability of cost assessments and motivates a need for more localized studies. Control costs-per-tonne for PM are typically highest except for mobile sources, for which NO_X_ emissions controls are most costly. The mix of sources, the pollution-generating equipment employed, and the target level of abatement all influence total and marginal abatement costs. Pollution control technologies, with multiple types available for a vast array of sources and pollutants, can also affect multiple pollutants at once, thus offering opportunities for total cost reduction. Pollution control technologies for most sources are mature and their costs are well established; still, their performance and costs vary widely across technologies and sources. This means that they must be evaluated in the context of the Saudi Arabian climate and the country’s currently installed emissions-generating technologies.

Evaluations of technologies to address shipping pollution were less prevalent than evaluations of other sources in the literature we reviewed, but fuel switching to lower-sulfur alternatives is a common control approach. Policy studies have shown that the effectiveness of ECAs can be undermined by adjacent, non-participating ports. This issue may be particularly acute for Saudi Arabia’s ports, as vessel traffic to and from neighboring oil-exporting states on the Gulf and through the Red Sea is substantial. For eastern ports, the Gulf Coordinating Council may offer a valuable policy venue for policy harmonization, which can be an important aspect of policy design for mobile emissions sources.

Policy makers face an array of options for incentivizing pollution mitigation. Among them are taxes and subsidies, command-and-control policies, cap-and-trade policies, and non-pecuniary and non-remunerative policies such as information alert systems—these last policies are particularly relevant for households and individuals. In evaluating costs and benefits localized to Saudi Arabia, evaluating both technology and policy costs is essential. Local pollution control evaluations must first consider technology costs to establish the abatement potential at each cost for each source type and location considered. Second, policy cost evaluations will establish which of the technologies are economical to deploy and how market incentives and outcomes will determine total costs of pollution control. In evaluating our second research question on mitigation cost effectiveness, we found that, though the literature is relatively more abundant in technology cost assessment studies, policy costs—particularly for larger pollution control efforts that span multiple sectors—may diverge significantly and therefore require independent evaluation. Indeed, the policy studies identified measured higher costs than the corresponding technology costs summarized from the gray literature.

The total cost effectiveness of pollution mitigation depends on both the technology options available, how operators of emissions sources are induced to implement those technologies, and the opportunity costs of doing so. To address our second research question in full, we also reviewed studies of broader policy costs of pollution control finding that costs differ from technology costs in a few ways. Policy costs consider broader economic impacts from implementing pollution controls, which can lead to higher cost estimates, but they also typically exclude the most expensive technology costs in all but the most stringent policies. Examples of broader economic impacts include reduced output, increased costs to consumers, opportunity costs associated with investment diverted to other uses (i.e., pollution control), and the effect of interacting taxes on economic efficiency losses. Few studies identified in our literature review explicitly and comprehensively addressed these costs through economic simulation or other modeling approaches. Strict engineering cost estimates were common, representing the least-cost technology approach to achieving given emissions targets (e.g., Lai et al., 2020; S. Zhang et al., 2021). Relatively few studies employed economy-wide, general equilibrium approaches capable of capturing the full interaction of markets, producers, consumers, and governments in an economically consistent fashion. Such approaches are especially important for mitigation policies that may induce larger economic disruptions, but economy-wide simulations are often challenged by the techno-economic heterogeneity and complexity of pollution controls across many sources.

### 4.3 Policy and practical implications

The effectiveness of adaptation options at reducing pollution exposures suggests that policies that provide actionable information (e.g., air quality alerts) and that subsidize required resources (e.g., equipment) could provide cost-effective environmental public health improvements in Saudi Arabia. Still, our literature review did not identify robust cost-benefit evaluations of environmental public health adaptation policies, and the centrality of behavioral responses in determining the effectiveness of such policies suggests that existing studies may not generalize well to other populations. Behavioral responses include populations’ responsiveness to air quality information; their financial capacity to invest in air quality equipment; their willingness to invest given how they value and discount future air quality benefits against up-front investment costs; and their ability and willingness to operate air quality equipment at optimal efficiency. For example, face masks may be excessively hot to wear outdoors in Saudi Arabia’s climate, separating pedestrian spaces from vehicular traffic may be costly given the country’s current infrastructure, and such separation may also be inconvenient or otherwise undesirable for pedestrians.

The technology literature for controlling emissions from electricity generation is rich and mature, though much of the control cost information resides in technical reports and public databases. Control technologies for oil- and natural gas–fired generators are particularly important to Saudi Arabia’s electricity sector, but control cost information for oil generation may be less rich than for coal and gas generation, given its minor role in electricity generation in most higher-income countries; furthermore, Saudi-specific performance and cost evaluations are still needed. Fuel switching is another valuable mitigation alternative to emissions control, and Saudi Arabian renewable resources and nuclear generation potential offer substantial opportunity for zero-emissions generation here. Policy studies for controlling pollution from electricity abound as the electricity sector is a major source of multiple pollutant emissions. The literature on multipollutant emissions suggests that there are often substantial co-benefits of pollution control when other emissions remain uncontrolled, but that direct regulation of the targeted pollutant is most cost effective.

The policy cost literature generally supports direct control of pollutants as more cost-effective than indirect control, either through co-benefits or through subsidies for clean alternatives. Policy co-benefits are significant, however, and should be considered in policy evaluations. As with technology costs, significant efficiency gains may be achievable through multipollutant control strategies, but their evaluation will depend heavily on the mix of specific sources, equipment, pollutants, and emissions targets relevant in Saudi Arabia. Though the literature generally favors more flexible mitigation policies that allow the lowest mitigation cost options to be deployed, policy design must also consider how the spatial and temporal distribution of emissions contribute to ambient concentrations in Saudi Arabia as well as how the location of exposed populations determines abatement benefits. Even where policy costs and benefits can be designed optimally to reflect spatio-temporal heterogeneity, policy makers must still consider efficiency-equity tradeoffs for the resulting benefit distributions along with the administrative practicality of implementing and enforcing highly nuanced policies.

Behavioral considerations for residential policy uptake and compliance, which are relevant for both of our research questions on mitigation and adaptation, may vary more by region than private sector adoption, which will be motivated more strictly by cost management considerations. Behavioral responses that differ from expectations can significantly undermine policy effectiveness, and such responses risk sub-optimal pollution mitigation and adaptation investments. Uncertainty may also be a significant factor in policy effectiveness in two ways. First, uncertainty in future private and public policy benefits may lead to heavier discounting of those benefits, undermining investment incentives. Second, uncertainty in benefit estimates may mean that, even with optimal *ex ante* investment, *ex post* realizations of benefits that differ significantly from expectations make for sub-optimal outcomes (e.g., too little or too much investment, or the wrong distribution thereof).

### 4.4 Limitations

Our literature reviews identified effective options for mitigating and adapting to air pollution. We examined the literature returned in our searches for their ability to address specific interventions and their efficiency, pollutant specificity, and regional relevance for Saudi Arabia. Environmental public health is a necessarily inter-disciplinary area of study, with different disciplines contributing to the science under different terminology. We approached this review from the perspective of economics and public health. The search terms we chose may have excluded relevant research on the topic. Indeed, certain relevant research articles we were aware of was not returned in our searches. As a result, there is undoubtedly more evidence available in the literature than we were able to consider in this review. There exists a body of technology cost literature beyond our search window, much of which is still regarded as reliable where technology has not changed appreciably, that would have been excluded by our time criterion or because it is part of a body of gray literature that we did not search.

Our exclusion criteria were designed to identify research that provided specific, quantitative estimates of the efficiency of specific mitigation or adaptation interventions for specific pollutants. There likely exists theoretical work in engineering, economics, behavioral sciences, epidemiology, and other disciplines that may have been returned in our search results but would not have passed our selection criteria. We elected to focus on specific, quantifiable research for tractability.

Among the research we did review, the findings we compiled may not be fully generalizable to the Saudi Arabian context. For example, we note in our review that behavioral responses, existing infrastructure, and differences in economic costs may vary significantly enough across location to drive markedly different results in Saudi Arabia. These differences could potentially be great enough to weaken the generalizability of even the higher-level findings we identify.

## 5 Conclusion and future perspectives

Significant opportunity exists for improving environmental public health in Saudi Arabia relative to the standards enjoyed by its peers by income. While existing international guidelines such as those from the WHO provide a helpful reference point for ambient air quality goals, they lack the cost and performance specificity for technologies and policies needed to address the air quality challenges Saudi Arabia faces. To address this gap, this review focused on the level and determinants of the effectiveness of mitigation and adaptation options for addressing environmental public health. We reviewed approximately 3,000 peer-reviewed publications relevant for improving environmental public health outcomes in Saudi Arabia. Our literature review identified research with effectiveness evaluations of specific pollution control interventions relevant to Saudi Arabia. Our review focused on technologies and policies available for mitigating and adapting to some of the most prominent air pollutants relevant to environmental public health: PM, NO_X_, and SO_2_.

Pollution mitigation technologies are myriad and, while they may be thoroughly studied and demonstrated as effective, must be evaluated in the Saudi context. Technology costs also vary widely. The specific costs that apply to Saudi Arabia must be determined through local engineering evaluations of existing and potential new sources. Policies that allow for flexibility in who mitigates their emissions will generally provide first-best options for cost minimization; however, important spatio-temporal heterogeneity exists in benefits and distributional equity considerations that must be factored into policy design. For the pollution that is not mitigated, several important adaptation strategies exist for creating less polluted indoor environments, filtering outdoor air with face masks, avoiding outdoor areas with especially hazardous pollution levels, and raising public awareness of pollution hazards when and where they exist. The literature suggests that these adaptation measures are effective at reducing exposure, but their cost-effectiveness requires further study with local relevance. Saudi-specific evaluations of adaptation measures may be especially important, as many local determinants of adaptation effectiveness exist, including behavioral responses and local infrastructure.

Our review found that adaptation has the least available information, especially with respect to cost effectiveness evaluations. We also found relatively few relevant studies on technology cost-effectiveness. While this may be partly the result of the “durability” of older technical reports and studies, up-to-date, local information remains a necessity for new environmental public health initiatives in locations that have less pollution control history such as Saudi Arabia. The policy cost evaluations identified several different important aspects of policy design but offered relatively little on optimal multipollutant policy design. While highly relevant for broadscale environmental public health efforts, such research may be primarily theoretical and therefore would have been excluded by our literature review for lack of specificity.

Future research evaluating specific multipollutant outcomes from policy interventions in diverse settings would serve this literature well. While techno-economic, economy-wide simulation modeling can be very informative, it is an incomplete substitute for empirical evidence of policy effectiveness. Still, richer simulations of diverse physical and techno-economic systems can greatly enhance policy evaluations by combining atmospheric modeling, spatially rich information on infrastructure and exposed populations, and multisectoral techno-economic characterization of pollution-generating activities. Such studies remain scarce and geographically under-diversified. Finally, behavioral studies that can account for individual and institutional biases in responding to policy initiatives over a greater range of socio-demographic diversity and with local relevance to countries expected to significantly expand their pollution control regimes such as Saudi Arabia can greatly enhance our understanding of policy effectiveness.

## Declarations

### Author contribution statement

All authors listed have significantly contributed to the development and the writing of this article.

### Funding statement

This research did not receive any specific grant from funding agencies in the public, commercial, or not-for-profit sectors.

### Data availability statement

Data included in article/supp. material/referenced in article.

### Declaration of interest’s statement

The authors declare no conflict of interest.

### Additional information

No additional information is available for this paper.

## Appendix B Search Terms

A1. Search parameters

All of the searches utilized similar filters and basic criteria to maintain consistency across the searches.

**Databases included**

• Web of Science: https://apps.webofknowledge.com/
• PubMed: https://pubmed.ncbi.nlm.nih.gov/

**Languages**

• English

**Document Types**

• Web of Science- “Article”
• PubMed – “Journal Article”

**Timespan**

• 2011 to 2021

A2. Web of Science

1) Intermediate Adaptation Interventions
  a) PM
    i) (ALL = ((PM2.5 or “PM 2.5” or PM10 or “PM 10” or “Particulate Matter”) and (alzheimer∗) and (polic∗) and (cost∗))
    ii) (ALL = ((PM2.5 or “PM 2.5” or PM10 or “PM 10” or “Particulate Matter”) and (“All-cause mortality” or mortality or death or “Cause of death”) and (polic∗) and (cost∗))
    iii) (ALL = ((PM2.5 or “PM 2.5” or PM10 or “PM 10” or “Particulate Matter”) and (morbidity) and (polic∗) and (cost∗))
    iv) (ALL=((PM2.5 or “PM 2.5” or PM10 or “PM 10” or “Particulate Matter”) and (Dementia) and (polic∗) and (cost∗))
    v) (ALL = ((PM2.5 or “PM 2.5” or PM10 or “PM 10” or “Particulate Matter”) and (Depression) and (polic∗) and (cost∗))
    vi) (ALL = ((PM2.5 or “PM 2.5” or PM10 or “PM 10” or “Particulate Matter”) and (“Cancer mortality” or cancer) and (polic∗) and (cost∗))
    vii) (ALL = ((PM2.5 or “PM 2.5” or PM10 or “PM 10” or “Particulate Matter”) and (Stroke) and (polic∗) and (cost∗))
    viii) (ALL = ((PM2.5 or “PM 2.5” or PM10 or “PM 10” or “Particulate Matter”) and (Parkinson∗) and (polic∗) and (cost∗))
    ix) (ALL = ((PM2.5 or “PM 2.5” or PM10 or “PM 10” or “Particulate Matter”) and (Asthma) and (polic∗) and (cost∗))
    x) (ALL = ((PM2.5 or “PM 2.5” or PM10 or “PM 10” or “Particulate Matter”) and (“Autistic syndrome disorder” or “Autism spectrum disorder” or “Autism” or “Autistic” or “Spectrum disorder”) and (polic∗) and (cost∗))
    xi) (ALL = ((PM2.5 or “PM 2.5” or PM10 or “PM 10” or “Particulate Matter”) and (suicide) and (polic∗) and (cost∗))
    xii) (ALL = ((PM2.5 or “PM 2.5” or PM10 or “PM 10” or “Particulate Matter”) and (pneumonia) and (polic∗) and (cost∗))
    xiii) (ALL = ((PM2.5 or “PM 2.5” or PM10 or “PM 10” or “Particulate Matter”) and (“Type 2 diabetes”) and (polic∗) and (cost∗))
    xiv) (ALL = ((PM2.5 or “PM 2.5” or PM10 or “PM 10” or “Particulate Matter”) and (“Incidence of coronary events” or “coronary events” or “coronary”) and (polic∗) and (cost∗))
  b) Ozone
    i) (ALL = ((Ozone or O3) and (“Preterm birth”) and (polic∗) and (cost∗))
    ii) (ALL = ((Ozone or O3) and (Parkinson∗) and (polic∗) and (cost∗))
    iii) (ALL = ((Ozone or O3) and (pneumonia) and (polic∗) and (cost∗))
  c) NO2
    i) ALL = ((NO2 or “Nitrogen Dioxide”) and (“Chronic kidney disease” or “kidney disease”) and (polic∗) and (cost∗))
    ii) (ALL = ((NO2 or “Nitrogen Dioxide”) and (“Respiratory mortality” or “respiratory morbidity” or respiratory) and (polic∗) and (cost∗))
    iii) (ALL = ((NO2 OR “Nitrogen Dioxide”) and (Asthma) and (polic∗) and (cost∗))
    iv) (ALL=((NO2 OR “Nitrogen Dioxide”) and (“cancer mortality” or cancer) and (polic∗) and (cost∗))
    v) (All = ((NO2 OR “Nitrogen Dioxide”) and (“Low birth weight”) and (polic∗) and (cost∗))
    vi) (ALL = ((NO2 OR “Nitrogen Dioxide”) and (“Pneumonia”) and (polic∗) and (cost∗))
    vii) (ALL = ((NO2 OR “Nitrogen Dioxide”) and (“Type 2 diabetes” or diabetes) and (polic∗) and (cost∗))
  d) SO2
    i) (ALL = ((SO2 OR “Sulfur Dioxide”) and (“Gestational diabetes mellitus” or “gestational” or “diabetes”) and (polic∗) and (cost∗))
    ii) (ALL = ((SO2 OR “Sulfur Dioxide”) and (“Pneumonia”) and (polic∗) and (cost∗))
  e) Desert Dust
    i) (ALL = ((“desert dust”) and (desert or “sand storm” or sand or dust) and (health∗ or “well-being” or medic∗ or wellness) and (cost∗) and (polic∗))
1) Adaptation Interventions
  b) Air Quality Alerts
    i) ALL = ((“air quality alert∗” or “air quality forecast”) AND (“particulate matter” or “PM2.5” or “PM 2.5” or “PM10” or “PM 10” or “ozone” or “O3” or “nitrogen dioxide” or “NO2” or “sulfur dioxide” or “SO2” or “desert dust”) AND (“cost∗”) AND (“polic∗”))
    ii) ALL = ((“air quality alert∗” or “air quality forecast”) AND (“particulate matter” or “PM2.5” or “PM 2.5” or “PM10” or “PM 10” or “ozone” or “O3” or “nitrogen dioxide” or “NO2” or “sulfur dioxide” or “SO2” or “desert dust”) AND (“polic∗”) AND (“effect∗”))
  c) Air Quality Index
    i) ALL = ((“air quality inde∗” or “air quality health inde∗”) AND (“particulate matter” or “PM2.5” or “PM 2.5” or “PM10” or “PM 10” or “ozone” or “O3” or “nitrogen dioxide” or “NO2” or “sulfur dioxide” or “SO2” or “desert dust”) AND (“cost∗”) AND (“polic∗”))
    ii) ALL = ((“air quality inde∗” or “air quality health inde∗”) AND (“particulate matter” or “PM2.5” or “PM 2.5” or “PM10” or “PM 10” or “ozone” or “O3” or “nitrogen dioxide” or “NO2” or “sulfur dioxide” or “SO2” or “desert dust”) AND (“polic∗”) AND (“effect∗”))
  d) Stay indoors
    i) ALL = ((“stay∗ indoor∗”) AND (“particulate matter” or “PM2.5” or “PM 2.5” or “PM10” or “PM 10” or “ozone” or “O3” or “nitrogen dioxide” or “NO2” or “sulfur dioxide” or “SO2” or “desert dust”) AND (“cost∗”) AND (“polic∗”))
    ii) ALL = ((“stay∗ indoor∗”) AND (“particulate matter” or “PM2.5” or “PM 2.5” or “PM10” or “PM 10” or “ozone” or “O3” or “nitrogen dioxide” or “NO2” or “sulfur dioxide” or “SO2” or “desert dust”) AND (“polic∗”) AND (“effect∗”))
  e) Close Windows
    i) ALL = ((“clos∗ window∗” or “window∗ clos∗”) AND (“particulate matter” or “PM2.5” or “PM 2.5” or “PM10” or “PM 10” or “ozone” or “O3” or “nitrogen dioxide” or “NO2” or “sulfur dioxide” or “SO2” or “desert dust”) AND (“cost∗”) AND (“polic∗”))
    ii) ALL = ((“clos∗ window∗” or “window∗ clos∗”) AND (“particulate matter” or “PM2.5” or “PM 2.5” or “PM10” or “PM 10” or “ozone” or “O3” or “nitrogen dioxide” or “NO2” or “sulfur dioxide” or “SO2” or “desert dust”) AND (“polic∗”) AND (“effect∗”))
  f) Avoid Exercise Outdoors
    i) ALL = ((“exercis∗”) AND (“outdoor∗”) AND (“particulate matter” or “PM2.5” or “PM 2.5” or “PM10” or “PM 10” or “ozone” or “O3” or “nitrogen dioxide” or “NO2” or “sulfur dioxide” or “SO2” or “desert dust”) AND (“cost∗”) AND (“polic∗”))
    ii) ALL = ((“exercis∗”) AND (“outdoor∗”) AND (“particulate matter” or “PM2.5” or “PM 2.5” or “PM10” or “PM 10” or “ozone” or “O3” or “nitrogen dioxide” or “NO2” or “sulfur dioxide” or “SO2” or “desert dust”) AND (“polic∗”) AND (“effect∗”))
  g) Individualized risk assessments
    i) ALL = ((“Individualized risk assessment∗” or “susceptible population∗” or “high risk population∗”) AND (“particulate matter” or “PM2.5” or “PM 2.5” or “PM10” or “PM 10” or “ozone” or “O3” or “nitrogen dioxide” or “NO2” or “sulfur dioxide” or “SO2” or “desert dust”) AND (“cost∗”) AND (“polic∗”))
    ii) ALL = ((“Individualized risk assessment∗” or “susceptible population∗” or “high risk population∗”) AND (“particulate matter” or “PM2.5” or “PM 2.5” or “PM10” or “PM 10” or “ozone” or “O3” or “nitrogen dioxide” or “NO2” or “sulfur dioxide” or “SO2” or “desert dust”) AND (“cost∗”) AND (“polic∗”) AND (“effectiv∗”))
  h) Indoor air filtering and portable air cleaners
    i) ALL = ((“indoor air filter∗” or “filter∗” or “filtration” or “air cleaner∗” or “HEPA” or “vent∗”) AND (“particulate matter” or “PM2.5” or “PM 2.5” or “PM10” or “PM 10” or “ozone” or “O3” or “nitrogen dioxide” or “NO2” or “sulfur dioxide” or “SO2” or “desert dust”) AND (“cost∗”) AND (“polic∗”))
    ii) ALL = ((“indoor air filter∗” or “filter∗” or “filtration” or “air cleaner∗” or “HEPA” or “vent∗”) AND (“particulate matter” or “PM2.5” or “PM 2.5” or “PM10” or “PM 10” or “ozone” or “O3” or “nitrogen dioxide” or “NO2” or “sulfur dioxide” or “SO2” or “desert dust”) AND (“polic∗”) AND (“effectiv∗”))
  i) Vehicle Ventilation or filtration
    i) ALL = ((“vehicle”) AND (“filtra∗” or “vent∗”) AND (“particulate matter” or “PM2.5” or “PM 2.5” or “PM10” or “PM 10” or “ozone” or “O3” or “nitrogen dioxide” or “NO2” or “sulfur dioxide” or “SO2” or “desert dust”) AND (“polic∗”) AND (“cost∗”))
    ii) ALL = ((“vehicle”) AND (“filtra∗” or “vent∗”) AND (“particulate matter” or “PM2.5” or “PM 2.5” or “PM10” or “PM 10” or “ozone” or “O3” or “nitrogen dioxide” or “NO2” or “sulfur dioxide” or “SO2” or “desert dust”) AND (“polic∗”) AND (“effect∗”))
  j) Avoid high trafficked routes
    i) (ALL = ((“route∗”) AND (“particulate matter” or “PM2.5” or “PM 2.5” or “PM10” or “PM 10” or “ozone” or “O3” or “nitrogen dioxide” or “NO2” or “sulfur dioxide” or “SO2” or “desert dust”) AND (“cost∗”) AND (“polic∗”))
    ii) (ALL = ((“route∗”) AND (“particulate matter” or “PM2.5” or “PM 2.5” or “PM10” or “PM 10” or “ozone” or “O3” or “nitrogen dioxide” or “NO2” or “sulfur dioxide” or “SO2” or “desert dust”) AND (“polic∗”) AND (“effectiv∗”))
  k) Masks, respirators
    i) ALL = ((“mask∗” or “facemask” or “personal filt∗” or “respirator∗” or “barrier method∗” or “pollution dome∗”) AND (“particulate matter” or “PM2.5” or “PM 2.5” or “PM10” or “PM 10” or “ozone” or “O3” or “nitrogen dioxide” or “NO2” or “sulfur dioxide” or “SO2” or “desert dust”) AND (“cost∗”) AND (“polic∗”))
    ii) ALL = ((“mask∗” or “facemask” or “personal filt∗” or “respirator∗” or “barrier method∗” or “pollution dome∗”) AND (“particulate matter” or “PM2.5” or “PM 2.5” or “PM10” or “PM 10” or “ozone” or “O3” or “nitrogen dioxide” or “NO2” or “sulfur dioxide” or “SO2” or “desert dust”) AND (“polic∗”) AND (“effect∗”))
  l) General
    i) ALL = ((“personal exposure” or “behavior chang∗” or “risk reduction∗” or “exposure reduction∗”) AND (“particulate matter” or “PM2.5” or “PM 2.5” or “PM10” or “PM 10” or “ozone” or “O3” or “nitrogen dioxide” or “NO2” or “sulfur dioxide” or “SO2” or “desert dust”) AND (“polic∗”) AND (“cost∗”))
    ii) ALL = ((“personal exposure” or “behavior chang∗” or “risk reduction∗” or “exposure reduction∗”) AND (“particulate matter” or “PM2.5” or “PM 2.5” or “PM10” or “PM 10” or “ozone” or “O3” or “nitrogen dioxide” or “NO2” or “sulfur dioxide” or “SO2” or “desert dust”) AND (“polic∗”) AND (“effectiv∗”))
2) Mitigation Technologies
  a) VOC
    i) (ALL =((“VOC∗” or “volatile organic compound∗”) and (“O3” or “ozone”) and (construction or “plant∗” or industr∗ or transportation or automobile∗ or ship∗ or vehicle∗ or boiler∗ or refiner∗ or “fossil fuels” or combustion or “off road equipment” or extract∗ or metal∗ or process∗ or smelt∗ or boiler∗ or turbin∗or “cement kiln∗” or “energy” or “agriculture” or “waste management”) and (technolog∗) and (“air quality” or “air pollution”) and (cost∗) and (abat∗ or reduc∗ or mitigat∗ or control∗))
  b) PM
    i) (ALL = ((PM2.5 or “PM 2.5” or PM10 or “PM 10” or Particulate Matter) and (construction or “plant∗” or industr∗ or transportation or automobile∗ or ship∗ or vehicle∗ or boiler∗ or refiner∗ or “fossil fuels” or combustion) and (technolog∗) and (“air quality” or “air pollution”) and (cost∗) and (abat∗ or reduc∗ or mitigat∗ or control∗))
  c) NO2
    i) (ALL = ((NO2 or “Nitrogen Dioxide”) and (“fossil fuel∗” or transportation or automobile∗ or ship∗ or vehicle∗ or plant∗ or “off road equipment” or “construction” or “industrial process∗”) and (“air quality” or “air pollution”) and (technolog∗) and (cost∗) and (abat∗ or reduc∗ or mitigat∗ or control∗))
  d) SO2
    i) (ALL = ((“SO2” or “Sulfur Dioxide”) and (“fossil fuel∗” or transportation or automobile∗ or ship∗ or vehicle∗ or plant∗ or extract∗ or metal∗ or process∗ or smelt∗ or construction or industr∗) and (“air quality” or “air pollution”) and (cost∗) and (abat∗ or reduc∗ or mitigat∗ or control∗) and (technolog∗))
  e) NOX & Ozone
    i) (ALL = ((NOX or “Nitrogen Oxide”) and (O3 or “ozone”) and (“fossil fuel∗” or transportation or automobile∗ or ship∗ or vehicle∗ or plant∗ or construction or industr∗ or boiler∗ or turbin∗or “cement kiln∗”) and (“air quality” or “air pollution”) and (cost∗) and (abat∗ or reduc∗ or mitigat∗ or control∗) and (technolog∗))
  f) Black Carbon
    i) (ALL = ((“black carbon”) and (“fossil fuel∗” or transportation or automobile∗ or ship∗ or vehicle∗ or plant∗ or construction or industr∗ or “household energy” or “agriculture” or “waste management”) and (“air quality” or “air pollution”) and (cost∗) and (abat∗ or reduc∗ or mitigat∗ or control∗) and (technolog∗))
  g) Desert Dust
    i) (ALL = ((“desert dust”) and (desert or “sand storm” or sand or dust) and (“air quality” or “air pollution”) and (cost∗) and (abat∗ or reduc∗ or mitigat∗ or control∗) and (technolog∗))
3) Mitigation Policies
  a) VOC
    i) (ALL = ((“VOC∗” or “volatile organic compound∗”) and (“O3” or “ozone”) and (construction or “plant∗” or industr∗ or transportation or automobile∗ or ship∗ or vehicle∗ or boiler∗ or refiner∗ or “fossil fuels” or combustion or “off road equipment” or extract∗ or metal∗ or process∗ or smelt∗ or boiler∗ or turbin∗or “cement kiln∗” or “energy” or “agriculture” or “waste management”) and (polic∗) and (“air quality” or “air pollution”) and (cost∗) and (abat∗ or reduc∗ or mitigat∗ or control∗))
  b) PM
    i) (ALL = ((PM2.5 or “PM 2.5” or PM10 or “PM 10” or Particulate Matter) and (construction or “plant∗” or industr∗ or transportation or automobile∗ or ship∗ or vehicle∗ or boiler∗ or refiner∗ or “fossil fuels” or combustion) and (polic∗) and (“air quality” or “air pollution”) and (cost∗) and (abat∗ or reduc∗ or mitigat∗ or control∗))
  c) NO2
    i) (ALL = ((NO2 or “Nitrogen Dioxide”) and (“fossil fuel∗” or transportation or automobile∗ or ship∗ or vehicle∗ or plant∗ or “off road equipment” or “construction” or “industrial process∗”) and (“air quality” or “air pollution”) and (polic∗) and (cost∗) and (abat∗ or reduc∗ or mitigat∗ or control∗))
  d) SO2
    i) (ALL = ((“SO2” or “Sulfur Dioxide”) and (“fossil fuel∗” or transportation or automobile∗ or ship∗ or vehicle∗ or plant∗ or extract∗ or metal∗ or process∗ or smelt∗ or construction or industr∗) and (“air quality” or “air pollution”) and (cost∗) and (abat∗ or reduc∗ or mitigat∗ or control∗) and (polic∗))
  e) NOX & Ozone
    i) (ALL = ((NOX or “Nitrogen Oxide”) and (O3 and “ozone”) and (“fossil fuel∗” or transportation or automobile∗ or ship∗ or vehicle∗ or plant∗ or construction or industr∗ or boiler∗ or turbin∗or “cement kiln∗”) and (“air quality” or “air pollution”) and (cost∗) and (abat∗ or reduc∗ or mitigat∗ or control∗) and (polic∗))
  f) Black Carbon
    i) (ALL = ((“black carbon”) and (“fossil fuel∗” or transportation or automobile∗ or ship∗ or vehicle∗ or plant∗ or construction or industr∗ or “household energy” or “agriculture” or “waste management”) and (“air quality” or “air pollution”) and (cost∗) and (abat∗ or reduc∗ or mitigat∗ or control∗) and (polic∗))
  g) Desert Dust
    i) (ALL = ((“desert dust”) and (desert or “sand storm” or sand or dust) and (“air quality” or “air pollution”) and (cost∗) and (abat∗ or reduc∗ or mitigat∗ or control∗) and (polic∗))

A3. PubMed

1) Intermediate Adaptation Interventions
  a) PM
    i) (“Particulate Matter”[Mesh] or PM2.5 or “PM 2.5” or PM10 or “PM 10” or “Particulate Matter”) AND (“Costs and Cost Analysis”[Mesh]) AND “(“Alzheimer Disease”[Mesh])
    ii) (“Particulate Matter”[Mesh] or PM2.5 or “PM 2.5” or PM10 or “PM 10” or “Particulate Matter”)) AND (“Costs and Cost Analysis”[Mesh]) AND (“Asthma”[Mesh])
    iii) (“Particulate Matter”[Mesh] or PM2.5 or “PM 2.5” or PM10 or “PM 10” or “Particulate Matter”)) AND (“Costs and Cost Analysis”[Mesh]) AND (“Mortality”[Mesh])
    iv) (“Particulate Matter”[Mesh] or PM2.5 or “PM 2.5” or PM10 or “PM 10” or “Particulate Matter”)) AND (“Costs and Cost Analysis”[Mesh]) AND (“Morbidity”[Mesh])
    v) (“Particulate Matter”[Mesh] or PM2.5 or “PM 2.5” or PM10 or “PM 10” or “Particulate Matter”)) AND (“Costs and Cost Analysis”[Mesh]) AND (“Dementia”[Mesh])
    vi) (“Particulate Matter”[Mesh] or PM2.5 or “PM 2.5” or PM10 or “PM 10” or “Particulate Matter”)) AND (“Costs and Cost Analysis”[Mesh]) AND (“Depression”[Mesh])
    vii) (“Particulate Matter”[Mesh] or PM2.5 or “PM 2.5” or PM10 or “PM 10” or “Particulate Matter”)) AND (“Costs and Cost Analysis”[Mesh]) and (cancer∗)
    viii) (“Particulate Matter”[Mesh] or PM2.5 or “PM 2.5” or PM10 or “PM 10” or “Particulate Matter”)) AND (“Costs and Cost Analysis”[Mesh]) AND (“Stroke”[Mesh])
    ix) (“Particulate Matter”[Mesh] or PM2.5 or “PM 2.5” or PM10 or “PM 10” or “Particulate Matter”)) AND (“Costs and Cost Analysis”[Mesh]) AND (“Parkinson Disease”[Mesh])
    x) (“Particulate Matter”[Mesh] or PM2.5 or “PM 2.5” or PM10 or “PM 10” or “Particulate Matter”)) AND (“Costs and Cost Analysis”[Mesh]) AND (“Autism Spectrum Disorder”[Mesh])
    xi) (“Particulate Matter”[Mesh] or PM2.5 or “PM 2.5” or PM10 or “PM 10” or “Particulate Matter”)) AND (“Costs and Cost Analysis”[Mesh]) AND (“Suicide”[Mesh])
    xii) (“Particulate Matter”[Mesh] or PM2.5 or “PM 2.5” or PM10 or “PM 10” or “Particulate Matter”)) AND (“Costs and Cost Analysis”[Mesh]) AND (“Pneumonia”[Mesh])
    xiii) (“Particulate Matter”[Mesh] or PM2.5 or “PM 2.5” or PM10 or “PM 10” or “Particulate Matter”)) AND (“Costs and Cost Analysis”[Mesh]) AND (“Diabetes Mellitus, Type 2”[Mesh])
    xiv) (“Particulate Matter”[Mesh] or PM2.5 or “PM 2.5” or PM10 or “PM 10” or “Particulate Matter”)) AND (“Costs and Cost Analysis”[Mesh]) AND (“Heart”[Mesh])
  b) Ozone
    i) (“Costs and Cost Analysis”[Mesh]) AND (“Ozone”[Mesh] or “ozone” or “O3”) AND (“Premature Birth”[Mesh])
    ii) (“Costs and Cost Analysis”[Mesh]) AND (“Ozone”[Mesh] or “ozone” or “O3”) AND (“Parkinson Disease”[Mesh])
    iii) (“Costs and Cost Analysis”[Mesh]) AND (“Ozone”[Mesh] or “ozone” or “O3”) AND (“Pneumonia”[Mesh])
  c) NO2
    i) (“Nitrogen Dioxide”[Mesh] or NO2 or “Nitrogen Dioxide”) AND (“Costs and Cost Analysis”[Mesh]) AND (“Kidney Diseases”[Mesh])
    ii) (“Nitrogen Dioxide”[Mesh] or NO2 or “Nitrogen Dioxide”) AND (“Costs and Cost Analysis”[Mesh]) AND (“Respiratory System”[Mesh])
    iii) (“Nitrogen Dioxide”[Mesh] or NO2 or “Nitrogen Dioxide”) AND (“Costs and Cost Analysis”[Mesh]) AND (“Asthma”[Mesh])
    iv) (“Nitrogen Dioxide”[Mesh] or NO2 or “Nitrogen Dioxide”) AND (“Costs and Cost Analysis”[Mesh]) AND (cancer∗)
    v) (“Nitrogen Dioxide”[Mesh] or NO2 or “Nitrogen Dioxide”) AND (“Costs and Cost Analysis”[Mesh]) AND (“Birth Weight”[Mesh])
    vi) (“Nitrogen Dioxide”[Mesh] or NO2 or “Nitrogen Dioxide”) AND (“Costs and Cost Analysis”[Mesh]) AND (“Pneumonia”[Mesh])
    vii) (“Nitrogen Dioxide”[Mesh] or NO2 or “Nitrogen Dioxide”) AND (“Costs and Cost Analysis”[Mesh]) AND (“Diabetes Mellitus, Type 2”[Mesh])
  d) SO2
    i) (“Sulfur Dioxide”[Mesh] or SO2 or “Sulfur Dioxide”) AND (“Costs and Cost Analysis”[Mesh]) AND (“Diabetes, Gestational”[Mesh])
    ii) (“Sulfur Dioxide”[Mesh] or SO2 or “Sulfur Dioxide”) AND (“Costs and Cost Analysis”[Mesh]) AND (“Pneumonia”[Mesh])
  e) Desert Dust
    i) (“desert dust”) AND (“cost∗”)
2) Adaptation Interventions
  a) Air Quality Alerts
    i) “air quality alert∗” or “air quality forecast” AND (“particulate matter” or “PM2.5” or “PM 2.5” or “PM10” or “PM 10” or “ozone” or “O3” or “nitrogen dioxide” or “NO2” or “sulfur dioxide” or “SO2” or “desert dust”) AND (“cost∗”) AND (“polic∗”))
    ii) “air quality alert∗” or “air quality forecast” AND (“particulate matter” or “PM2.5” or “PM 2.5” or “PM10” or “PM 10” or “ozone” or “O3” or “nitrogen dioxide” or “NO2” or “sulfur dioxide” or “SO2” or “desert dust”) AND (“polic∗”) AND (“effect∗”))
  b) Air Quality Index
    i) (“air quality inde∗” or “air quality health inde∗”) AND (“particulate matter” or “PM2.5” or “PM 2.5” or “PM10” or “PM 10” or “ozone” or “O3” or “nitrogen dioxide” or “NO2” or “sulfur dioxide” or “SO2” or “desert dust”) AND (“cost∗”) AND (“polic∗”))
    ii) (“air quality inde∗” or “air quality health inde∗”) AND (“particulate matter” or “PM2.5” or “PM 2.5” or “PM10” or “PM 10” or “ozone” or “O3” or “nitrogen dioxide” or “NO2” or “sulfur dioxide” or “SO2” or “desert dust”) AND (“polic∗”) AND (“effect∗”))
  c) Stay indoors
    i) (“stay∗ indoor∗”) AND (“particulate matter” or “PM2.5” or “PM 2.5” or “PM10” or “PM 10” or “ozone” or “O3” or “nitrogen dioxide” or “NO2” or “sulfur dioxide” or “SO2” or “desert dust”) AND (“cost∗”) AND (“polic∗”))
    ii) (“stay∗ indoor∗”) AND (“particulate matter” or “PM2.5” or “PM 2.5” or “PM10” or “PM 10” or “ozone” or “O3” or “nitrogen dioxide” or “NO2” or “sulfur dioxide” or “SO2” or “desert dust”) AND (“polic∗”) AND (“effect∗”))
  d) Close Windows
    i) (“clos∗ window∗” or “window∗ clos∗”) AND (“particulate matter” or “PM2.5” or “PM 2.5” or “PM10” or “PM 10” or “ozone” or “O3” or “nitrogen dioxide” or “NO2” or “sulfur dioxide” or “SO2” or “desert dust”) AND (“cost∗”) AND (“polic∗”))
    ii) (“clos∗ window∗” or “window∗ clos∗”) AND (“particulate matter” or “PM2.5” or “PM 2.5” or “PM10” or “PM 10” or “ozone” or “O3” or “nitrogen dioxide” or “NO2” or “sulfur dioxide” or “SO2” or “desert dust”) AND (“polic∗”) AND (“effect∗”))
  e) Avoid Exercise Outdoors
    i) ALL= ((“outdoor∗ exercis∗” or “exercis∗ outdoor∗”) AND (“particulate matter” or “PM2.5” or “PM 2.5” or “PM10” or “PM 10” or “ozone” or “O3” or “nitrogen dioxide” or “NO2” or “sulfur dioxide” or “SO2” or “desert dust”) AND (“cost∗”) AND (“polic∗”))
    ii) ((“exercis∗”) AND (“outdoor∗”) AND (“particulate matter” or “PM2.5” or “PM 2.5” or “PM10” or “PM 10” or “ozone” or “O3” or “nitrogen dioxide” or “NO2” or “sulfur dioxide” or “SO2” or “desert dust”) AND (“polic∗”) AND (“effect∗”))
  f) Individualized risk assessments
    i) (“Individualized risk assessment∗” or “susceptible population∗” or “high risk population∗”) AND (“particulate matter” or “PM2.5” or “PM 2.5” or “PM10” or “PM 10” or “ozone” or “O3” or “nitrogen dioxide” or “NO2” or “sulfur dioxide” or “SO2” or “desert dust”) AND (“cost∗”) AND (“polic∗”))
    ii) (“Individualized risk assessment∗” or “susceptible population∗” or “high risk population∗”) AND (“particulate matter” or “PM2.5” or “PM 2.5” or “PM10” or “PM 10” or “ozone” or “O3” or “nitrogen dioxide” or “NO2” or “sulfur dioxide” or “SO2” or “desert dust”) AND (“polic∗”) AND (“effect∗”))
  g) Indoor air filtering and portable air cleaners
    i) (“indoor air filter∗” or “filter∗” or “filtration” or “air cleaner∗” or “HEPA” or “vent∗”) AND (“particulate matter” or “PM2.5” or “PM 2.5” or “PM10” or “PM 10” or “ozone” or “O3” or “nitrogen dioxide” or “NO2” or “sulfur dioxide” or “SO2” or “desert dust”) AND (“cost∗”) AND (“polic∗”))
    ii) (“indoor air filter∗” or “filter∗” or “filtration” or “air cleaner∗” or “HEPA” or “vent∗”) AND (“particulate matter” or “PM2.5” or “PM 2.5” or “PM10” or “PM 10” or “ozone” or “O3” or “nitrogen dioxide” or “NO2” or “sulfur dioxide” or “SO2” or “desert dust”) AND (“polic∗”) AND (“effect∗”))
  h) Vehicle Ventilation or filtration
    i) ((“vehicle”) AND (“filtra∗” or “vent∗”) AND (“particulate matter” or “PM2.5” or “PM 2.5” or “PM10” or “PM 10” or “ozone” or “O3” or “nitrogen dioxide” or “NO2” or “sulfur dioxide” or “SO2” or “desert dust”) AND (“cost∗”) AND (“polic∗”))
    ii) ((“vehicle”) AND (“filtra∗” or “vent∗”) AND (“particulate matter” or “PM2.5” or “PM 2.5” or “PM10” or “PM 10” or “ozone” or “O3” or “nitrogen dioxide” or “NO2” or “sulfur dioxide” or “SO2” or “desert dust”) AND (“polic∗”) AND (“effect∗”))
  i) Avoid high trafficked routes
    i) (route∗”) AND (“particulate matter” or “PM2.5” or “PM 2.5” or “PM10” or “PM 10” or “ozone” or “O3” or “nitrogen dioxide” or “NO2” or “sulfur dioxide” or “SO2” or “desert dust”) AND (“cost∗”) AND (“polic∗”))
    ii) (route∗”) AND (“particulate matter” or “PM2.5” or “PM 2.5” or “PM10” or “PM 10” or “ozone” or “O3” or “nitrogen dioxide” or “NO2” or “sulfur dioxide” or “SO2” or “desert dust”) AND (“polic∗”) AND (“effect∗”))
  j) Masks, respirators
    i) (“mask∗” or “facemask” or “personal filt∗” or “respirator∗” or “barrier method∗” or “pollution dome∗”) AND (“particulate matter” or “PM2.5” or “PM 2.5” or “PM10” or “PM 10” or “ozone” or “O3” or “nitrogen dioxide” or “NO2” or “sulfur dioxide” or “SO2” or “desert dust”) AND (“cost∗”) AND (“polic∗”))
    ii) (“mask∗” or “facemask” or “personal filt∗” or “respirator∗” or “barrier method∗” or “pollution dome∗”) AND (“particulate matter” or “PM2.5” or “PM 2.5” or “PM10” or “PM 10” or “ozone” or “O3” or “nitrogen dioxide” or “NO2” or “sulfur dioxide” or “SO2” or “desert dust”) AND (“polic∗”) AND (“effect∗”)
  k) General
    i) (“personal exposure” or “behavior chang∗” or “risk reduction∗” or “exposure reduction∗”) AND (“particulate matter” or “PM2.5” or “PM 2.5” or “PM10” or “PM 10” or “ozone” or “O3” or “nitrogen dioxide” or “NO2” or “sulfur dioxide” or “SO2” or “desert dust”) AND (“polic∗”) AND (“effect∗”)
    ii) (“personal exposure” or “behavior chang∗” or “risk reduction∗” or “exposure reduction∗”) AND (“particulate matter” or “PM2.5” or “PM 2.5” or “PM10” or “PM 10” or “ozone” or “O3” or “nitrogen dioxide” or “NO2” or “sulfur dioxide” or “SO2” or “desert dust”) AND (“polic∗”) AND (“cost∗”)
