# Supplementary material for: Effective options for addressing air quality– related environmental public health burdens in Saudi Arabia
Source: Heliyon. 2022 Aug 19;8(9):e10335. doi: 10.1016/j.heliyon.2022.e10335 (PMC9463589; doi:10.1016/j.heliyon.2022.e10335)
Supplement: Supplementary Materials [file mmc1.docx]

# Appendix C: Studies Considered in Analysis

**Adaption Interventions**

Alari, A., Schwarz, L., Zabrocki, L., Le Nir, G., Chaix, B., & Benmarhnia, T. 2021. The effects of an air quality alert program on premature mortality: A difference-in-differences evaluation in the region of Paris. *Environment International*, *156*, 106583. https://doi.org/10.1016/j.envint.2021.106583

Aldred, J. R., Darling, E., Morrison, G., Siegel, J., & Corsi, R. L. 2016. Benefit‐cost analysis of commercially available activated carbon filters for indoor ozone removal in single‐family homes. *Indoor Air*, *26*(3), 501-512. https://doi.org/10.1111/ina.12220

Gilliland, J., Maltby, M., Xu, X., Luginaah, I., Loebach, J., & Shah, T. 2019. Is active travel a breath of fresh air? Examining children's exposure to air pollution during the school commute. *Spatial and*  *spatio-temporal epidemiology*, *29*, 51-57. https://doi.org/10.1016/j.sste.2019.02.004

Hahm, Y., & Yoon, H. 2021. The impact of air pollution alert services on respiratory diseases: generalized additive modeling study in South Korea. *Environmental Research Letters*, *16*(6), 064048. DOI:10.1088/1748-9326/ac002f

Hong, A. 2019. Effect of mode shift from car to light rail on personal exposure: A controlled experiment. *Atmospheric Environment*, *196*, 53-65. https://doi.org/10.1016/j.atmosenv.2018.10.004

Jarjour, S., Jerrett, M., Westerdahl, D., de Nazelle, A., Hanning, C., Daly, L., Lipsitt, J. & Balmes, J. 2013. Cyclist route choice, traffic-related air pollution, and lung function: a scripted exposure study. *Environmental Health*, *12*(1), 1-12. https://doi.org/10.1186/1476-069X-12-14

Mason, T. G., Schooling, C. M., Chan, K. P., & Tian, L. 2019. An evaluation of the Air Quality Health Index Program on respiratory diseases in Hong Kong: an interrupted time series analysis. *Atmospheric Environment*, *211*, 151-158. https://doi.org/10.1016/j.atmosenv.2019.05.013

Mason, T. G., Schooling, C. M., Ran, J., Chan, K. P., & Tian, L. 2020. Does the AQHI reduce cardiovascular hospitalization in Hong Kong’s elderly population?. *Environment international*, *135*, 105344. https://doi.org/10.1016/j.envint.2019.105344

Patel, D., Shibata, T., Wilson, J., & Maidin, A. 2016. Challenges in evaluating PM concentration levels, commuting exposure, and mask efficacy in reducing PM exposure in growing, urban communities in a developing country. *Science of The Total Environment*, *543*, 416-424. https://doi.org/10.1016/j.scitotenv.2015.10.163

Paulin, L. M., Diette, G. B., Scott, M., McCormack, M. C., Matsui, E. C., Curtin‐Brosnan, J., Williams, D.L., Kidd-Taylor, A., Shea, P. N., & Hansel, N. N. 2014. Home interventions are effective at decreasing indoor nitrogen dioxide concentrations. *Indoor air*, *24*(4), 416-424. https://doi.org/10.1111/ina.12085

Tong, Z., Li, Y., Westerdahl, D., & Freeman, R. B. 2020. The impact of air filtration units on primary school students’ indoor exposure to particulate matter in China. *Environmental Pollution*, *266*, 115107. https://doi.org/10.1016/j.envpol.2020.115107

Zhan, Y., Johnson, K., Norris, C., Shafer, M. M., Bergin, M. H., Zhang, Y., Zhang, J., & Schauer, J. J. 2018. The influence of air cleaners on indoor particulate matter components and oxidative potential in residential households in Beijing. *Science of The Total Environment*, *626*, 507-518. https://doi.org/10.1016/j.scitotenv.2018.01.024

**Mitigation Policies**

Chen, F., Yamashita, K., Kurokawa, J., & Klimont, Z. 2015. Cost–Benefit Analysis of Reducing Premature Mortality Caused by Exposure to Ozone and PM 2.5 in East Asia in 2020. *Water, Air,*  *& Soil Pollution*, *226*(4), 1-17. https://doi.org/10.1007/s11270-015-2316-7

Chiesa, M., Perrone, M. G., Cusumano, N., Ferrero, L., Sangiorgi, G., Bolzacchini, E., Lorenzoni, A., & Denti, A. B. 2014. An environmental, economical and socio-political analysis of a variety of urban air-pollution reduction policies for primary PM10 and NOx: The case study of the Province of Milan (Northern Italy). *Environmental science & policy*, *44*, 39-50. https://doi.org/10.1016/j.envsci.2014.07.012

Fowlie, M., Knittel, C. R., & Wolfram, C. 2012. Sacred cars? Cost-effective regulation of stationary and nonstationary pollution sources. *American Economic Journal: Economic Policy*, *4*(1), 98-126. DOI: 10.1257/pol.4.1.98

Guo, X., Zhao, L., Chen, D., Jia, Y., Zhao, N., Liu, W., & Cheng, S. 2018. Air quality improvement and health benefit of PM 2.5 reduction from the coal cap policy in the Beijing–Tianjin–Hebei (BTH) region, China. *Environmental Science and Pollution Research*, *25*(32), 32709-32720. https://doi.org/10.1007/s11356-018-3014-y

Hasanbeigi, A., Lobscheid, A., Lu, H., Price, L., & Dai, Y. 2013. Quantifying the co-benefits of energy- efficiency policies: a case study of the cement industry in Shandong Province, China. *Science of*  *the total environment*, *458*, 624-636. https://doi.org/10.1016/j.scitotenv.2013.04.031

Howard, D. B., Thé, J., Soria, R., Fann, N., Schaeffer, R., & Saphores, J. D. M. 2019. Health benefits and control costs of tightening particulate matter emissions standards for coal power plants-The case of Northeast Brazil. *Environment international*, *124*, 420-430. https://doi.org/10.1016/j.envint.2019.01.029

Li, N., Chen, W., Rafaj, P., Kiesewetter, G., Schöpp, W., Wang, H., Zhang, H., Krey, V., & Riahi, K. 2019. Air quality improvement co-benefits of low-carbon pathways toward well below the 2 C climate target in China. *Environmental science & technology*, *53*(10), 5576-5584. DOI: 10.1021/acs.est.8b06948.

Lai, H. C., Hsiao, M. C., Liou, J. L., Lai, L. W., Wu, P. C., & Fu, J. S. 2020. Using Costs and Health Benefits to Estimate the Priority of Air Pollution Control Action Plan: A Case Study in Taiwan. *Applied Sciences*, *10*(17), 5970. https://doi.org/10.3390/app10175970

Liu, H., Meng, Z. H., Shang, Y., Lv, Z. F., Jin, X. X., Fu, M. L., & He, K. B. 2018. Shipping emission forecasts and cost-benefit analysis of China ports and key regions’ control. *Environmental*  *pollution*, *236*, 49-59. https://doi.org/10.1016/j.envpol.2018.01.018

Mardones, C., & Cabello, M. 2019. Effectiveness of local air pollution and GHG taxes: The case of Chilean industrial sources. *Energy Economics*, *83*, 491-500. https://doi.org/10.1016/j.eneco.2019.08.007

McDonald-Buller, E., Kimura, Y., Craig, M., McGaughey, G., Allen, D., & Webster, M. 2016. Dynamic Management of NO x and SO2 Emissions in the Texas and Mid-Atlantic Electric Power Systems and Implications for Air Quality. *Environmental science & technology*, *50*(3), 1611-1619. https://doi.org/10.1021/acs.est.5b04175

Peng, Z. S., Zhang, Y. L., Shi, G. M., & Chen, X. H. 2019. Cost and effectiveness of emissions trading considering exchange rates based on an agent-based model analysis. *Journal of Cleaner*  *Production*, *219*, 75-85. https://doi.org/10.1016/j.jclepro.2019.01.303

Pinchasik, D. R., Hovi, I. B., Mjøsund, C. S., Grønland, S. E., Fridell, E., & Jerksjö, M. 2020. Crossing borders and expanding modal shift measures: Effects on mode choice and emissions from freight transport in the Nordics. *Sustainability*, *12*(3), 894. https://doi.org/10.3390/su12030894

Raff, Z., & Walter, J. M. 2019. Evaluating the efficacy of ambient air quality standards at coal-fired power plants. *Available at SSRN 3335401*. http://dx.doi.org/10.2139/ssrn.3335401

Relvas, H., & Miranda, A. I. 2018. An urban air quality modeling system to support decision-making: design and implementation. *Air Quality, Atmosphere & Health*, *11*(7), 815-824. https://doi.org/10.1007/s11869-018-0587-z

Rodgers, M., Coit, D., Felder, F., & Carlton, A. 2019. Assessing the effects of power grid expansion on human health externalities. *Socio-Economic Planning Sciences*, *66*, 92-104. https://doi.org/10.1016/j.seps.2018.07.011

Sanderson, W., Striessnig, E., Schöpp, W., & Amann, M. 2013. Effects on well-being of investing in cleaner air in India. *Environmental science & technology*, *47*(23), 13222-13229. https://doi.org/10.1021/es402867r

Shih, Y. H., & Tseng, C. H. 2014. Cost-benefit analysis of sustainable energy development using life- cycle co-benefits assessment and the system dynamics approach. *Applied energy*, *119*, 57-66. https://doi.org/10.1016/j.apenergy.2013.12.031

Sun, L., Webster, M., McGaughey, G., McDonald-Buller, E. C., Thompson, T., Prinn, R., ... & Allen, D. T. 2012. Flexible NOx abatement from power plants in the eastern United States. *Environmental*  *science & technology*, *46*(10), 5607-5615. https://doi.org/10.1021/es204290s

Taksibi, F., Khajehpour, H., & Saboohi, Y. 2020. On the environmental effectiveness analysis of energy policies: A case study of air pollution in the megacity of Tehran. *Science of The Total Environment*, *705*, 135824. https://doi.org/10.1016/j.scitotenv.2019.135824

Wang, L., Patel, P. L., Yu, S., Liu, B., McLeod, J., Clarke, L. E., & Chen, W. 2016. Win–win strategies to promote air pollutant control policies and non-fossil energy target regulation in China. *Applied*  *energy*, *163*, 244-253. https://doi.org/10.1016/j.apenergy.2015.10.189

Wang, S., Qing, L. J., Wang, H., & Li, H. Y. 2018. Integrated assessment of environmental performance- based contracting for sulfur dioxide emission control in Chinese coal power plants. *Journal of*  *Cleaner Production*, *177*, 878-887. https://doi.org/10.1016/j.jclepro.2017.12.280

Wang, K., Wang, J., Hubacek, K., Mi, Z., & Wei, Y. M. 2020. A cost–benefit analysis of the environmental taxation policy in China: A frontier analysis‐based environmentally extended input– output optimization method. *Journal of Industrial Ecology*, *24*(3), 564-576. https://doi.org/10.1111/jiec.12947

Xiao, C., Chang, M., Guo, P., Chen, Q., & Tian, X. 2019. Comparison of the cost-effectiveness of eliminating high-polluting old vehicles and imposing driving restrictions to reduce vehicle emissions in Beijing. *Transportation Research Part D: Transport and Environment*, *67*, 291-302. https://doi.org/10.1016/j.trd.2018.10.006

Xu, X., Wang, Q., Hu, H., & Wang, X. 2021. Air Pollution Control: An Analysis of China’s Industrial Off- Peak Production Policy through the Quasi-Natural Experiment Method. *Sustainability*, *13*(9), 4808. https://doi.org/10.3390/su13094808

Xue, J., Zhao, L., Fan, L., & Qian, Y. 2015. An interprovincial cooperative game model for air pollution control in China. *Journal of the Air & Waste Management Association*, *65*(7), 818-827. https://doi.org/10.1080/10962247.2015.1021935

Zhou, J., Wang, J., Jiang, H., Cheng, X., Lu, Y., Zhang, W., Bi, J., Xue, W., & Liu, N. 2019. Cost-benefit analysis of yellow-label vehicles scrappage subsidy policy: A case study of Beijing-Tianjin- Hebei region of China. *Journal of Cleaner Production*, *232*, 94-103. https://doi.org/10.1016/j.jclepro.2019.05.312

**Mitigation Technologies**

Ammar, N. R., & Seddiek, I. S. 2017. Eco-environmental analysis of ship emission control methods: Case study RO-RO cargo vessel. *Ocean Engineering*, *137*, 166-173. https://doi.org/10.1016/j.oceaneng.2017.03.052

Chen, T. L., Fang, Y. K., Pei, S. L., Pan, S. Y., Chen, Y. H., & Chiang, P. C. 2019. Development and deployment of integrated air pollution control, CO2 capture and product utilization via a high- gravity process: comprehensive performance evaluation. *Environmental pollution*, *252*, 1464- 1475. https://doi.org/10.1016/j.envpol.2019.06.047

Evans, J. S., Rojas‐Bracho, L., Hammitt, J. K., & Dockery, D. W. 2021. Mortality benefits and control costs of improving air quality in Mexico City: The case of heavy duty diesel vehicles. *Risk*  *Analysis*, *41*(4), 661-677. https://doi.org/10.1111/risa.13655

Galvis, B., Bergin, M., Boylan, J., Huang, Y., Bergin, M., & Russell, A. G. 2015. Air quality impacts and health-benefit valuation of a low-emission technology for rail yard locomotives in Atlanta Georgia. *Science of the Total Environment*, *533*, 156-164. https://doi.org/10.1016/j.scitotenv.2015.06.064

Kanada, M., Dong, L., Fujita, T., Fujii, M., Inoue, T., Hirano, Y., Togawa, T., & Geng, Y. 2013. Regional disparity and cost-effective SO2 pollution control in China: A case study in 5 mega-cities. *Energy policy*, *61*, 1322-1331. https://doi.org/10.1016/j.enpol.2013.05.105

Li, H., Tan, X., Guo, J., Zhu, K., & Huang, C. 2019. Study on an implementation scheme of synergistic emission reduction of CO2 and air pollutants in China’s steel industry. *Sustainability*, *11*(2), 352. https://doi.org/10.3390/su11020352

Mardones, C., & Saavedra, A. 2016. Comparison of economic instruments to reduce PM2. 5 from industrial and residential sources. *Energy Policy*, *98*, 443-452. https://doi.org/10.1016/j.enpol.2016.09.011

Miranda, A. I., Ferreira, J., Silveira, C., Relvas, H., Duque, L., Roebeling, P., Lopes, M., Costa, S., Montiero,A., Gama, C., Sa, E., Borrego, C. & Teixeira, J. P. 2016. A cost-efficiency and health benefit approach to improve urban air quality. *Science of the Total Environment*, *569*, 342-351. https://doi.org/10.1016/j.scitotenv.2016.06.102

Nazar, R., Srinivasan, S. L., Kanudia, A., & Asundi, J. 2021. Implication of emission regulation on cost and tariffs of coal-based power plants in India: A system modelling approach. *Energy Policy*, *148*, 111924. https://doi.org/10.1016/j.enpol.2020.111924

Pinprateep, W., & Pharino, C. 2018. Emission intensity and abatement cost of fossil fuel power plants in Thailand. *Environmental Engineering & Management Journal (EEMJ)*, *17*(9).

Obara, S. Y., & Li, J. 2020. Evaluation of the introduction of a hydrogen supply chain using a conventional gas pipeline–A case study of the Qinghai–Shanghai hydrogen supply chain. *International Journal of Hydrogen Energy*, *45*(58), 33846-33859. https://doi.org/10.1016/j.ijhydene.2020.09.009

Ravina, M., Gamberini, C., Casasso, A., & Panepinto, D. 2020. Environmental and health impacts of domestic hot water (DHW) boilers in urban areas: a case study from Turin, NW Italy. *International*  *journal of environmental research and public health*, *17*(2), 595. https://doi.org/10.3390/ijerph17020595

Shawhan, D. L., & Picciano, P. D. 2019. Costs and benefits of saving unprofitable generators: A simulation case study for US coal and nuclear power plants. *Energy Policy*, *124*, 383-400. https://doi.org/10.1016/j.enpol.2018.07.040

Sun, J., Schreifels, J., Wang, J., Fu, J. S., & Wang, S. 2014. Cost estimate of multi-pollutant abatement from the power sector in the Yangtze River Delta region of China. *energy policy*, *69*, 478-488. https://doi.org/10.1016/j.enpol.2014.02.007

Tong, F., Hendrickson, C., Biehler, A., Jaramillo, P., & Seki, S. 2017. Life cycle ownership cost and environmental externality of alternative fuel options for transit buses. *Transportation Research*  *Part D: Transport and Environment*, *57*, 287-302. https://doi.org/10.1016/j.trd.2017.09.023

[dataset] United States Environmental Protection Agency (EPA). 2020. Menu of control measures for NAAQS implementation (MCM). https://www.epa.gov/air-quality-implementation-plans/menu-control-measures-naaqs-implementation

Wadud, Z., & Khan, T. 2013. Air quality and climate impacts due to CNG conversion of motor vehicles in Dhaka, Bangladesh. *Environmental science & technology*, *47*(24), 13907-13916. https://doi.org/10.1021/es402338b

Xu, F., Cui, F., & Xiang, N. 2021. Roadmap of green transformation for a steel-manufacturing intensive city in China driven by air pollution control. *Journal of Cleaner Production*, *283*, 124643. https://doi.org/10.1016/j.jclepro.2020.124643

Yang, X., Liu, H., Cui, H., Man, H., Fu, M., Hao, J., & He, K. 2015. Vehicular volatile organic compounds losses due to refueling and diurnal process in China: 2010–2050. *Journal of Environmental*  *Sciences*, *33*, 88-96. https://doi.org/10.1016/j.jes.2015.01.012

Zhang, S., Worrell, E., Crijns-Graus, W., Wagner, F., & Cofala, J. 2014. Co-benefits of energy efficiency improvement and air pollution abatement in the Chinese iron and steel industry. *Energy*, *78*, 333- 345. https://doi.org/10.1016/j.energy.2014.10.018

Zhang, W., Li, A., Xu, Y., & Liu, J. 2018. The theory-practice gap of black carbon mitigation technologies in rural China. *Atmospheric Environment*, *174*, 122-131. https://doi.org/10.1016/j.atmosenv.2017.11.050
